# Evolutionary stability, landscape heterogeneity, and human land‐usage shape population genetic connectivity in the Cape Floristic Region biodiversity hotspot

**DOI:** 10.1111/eva.13185

**Published:** 2021-01-13

**Authors:** Erica E. Tassone, Lindsay S. Miles, Rodney J. Dyer, Michael S. Rosenberg, Richard M. Cowling, Brian C. Verrelli

**Affiliations:** ^1^ School of Life Sciences Arizona State University Tempe Arizona USA; ^2^ Center for the Study of Biological Complexity Virginia Commonwealth University Richmond Virginia USA; ^3^ Center for Environmental Studies Virginia Commonwealth University Richmond Virginia USA; ^4^ African Centre for Coastal Palaeoscience Botany Department Nelson Mandela University Port Elizabeth South Africa

**Keywords:** Cape Floristic Region, gene flow, land use, social network, urbanization

## Abstract

As human‐induced change eliminates natural habitats, it impacts genetic diversity and population connectivity for local biodiversity. The South African Cape Floristic Region (CFR) is the most diverse extratropical area for plant biodiversity, and much of its habitat is protected as a UNESCO World Heritage site. There has long been great interest in explaining the underlying factors driving this unique diversity, especially as much of the CFR is endangered by urbanization and other anthropogenic activity. Here, we use a population and landscape genetic analysis of SNP data from the CFR endemic plant *Leucadendron salignum* or “common sunshine conebush” as a model to address the evolutionary and environmental factors shaping the vast CFR diversity. We found that high population structure, along with relatively deeper and older genealogies, is characteristic of the southwestern CFR, whereas low population structure and more recent lineage coalescence depict the eastern CFR. Population network analyses show genetic connectivity is facilitated in areas of lower elevation and higher seasonal precipitation. These population genetic signatures corroborate CFR species‐level patterns consistent with high Pleistocene biome stability and landscape heterogeneity in the southwest, but with coincident instability in the east. Finally, we also find evidence of human land‐usage as a significant gene flow barrier, especially in severely threatened lowlands where genetic connectivity has been historically the highest. These results help identify areas where conservation plans can prioritize protecting high genetic diversity threatened by contemporary human activities within this unique cultural UNESCO site.

## 1 INTRODUCTION

Loss of biodiversity through human‐induced habitat alteration has large impacts in reducing the sustainability of ecosystem services (Balmford & Bond, 2005; Flombaum & Sala, 2008; Sala et al., 2000). The impact of urbanization on biodiversity has received great attention (Alberti, 2015; Johnson & Munshi‐South, 2017; Miles et al., 2019; Rivkin et al., 2018), yet anthropogenic activities in general have likely disturbed all natural areas (Kareiva et al., 2007). Abrupt changes due to human land‐usage, including deforestation and agriculture, impact biodiversity long after recovery efforts (Jung et al., 2019; Moreno‐Mateos et al., 2017; Newbold et al., 2015). Management efforts indicate that local factors are the best predictors of biodiversity, but that multi‐scale landscape approaches are needed to marry historical and contemporary patterns with land‐usage change (Auffret et al., 2018; Gonthier et al., 2014).

The details of evolutionary population demography, such as gene flow and population structure across temporal and spatial scales, are key to understanding anthropogenic impact on long‐term biodiversity. As human‐fragmented landscapes reduce gene flow, species can be challenged by lower genetic diversity within populations and increased genetic differentiation among them (Keyghobadi, 2007; McKinney, 2006), which over long‐term can lead to increased inbreeding and local extinction (Charlesworth & Charlesworth, 1987; Frankham, 2005). While microevolutionary studies should directly incorporate anthropogenic impacts in modeling biodiversity dynamics (Alberti et al., 2020; Des Roches et al., 2020), how patterns of gene flow across both natural and anthropogenic features, especially across highly heterogeneous environments, influences conservation models is still a burgeoning field of research. Recent work emphasizes that challenges stem from the unknown effects of land‐usage from large agricultural development to small but dense urban areas, the patchy habitat structure of the landscape, the entangled historical and contemporary effects, and interactions among variables (Ballare & Jha, 2020; Centeno‐Cuadros et al., 2017; Fusco et al., 2020; Gonzalez‐Serna et al., 2019; Miles, Dyer, et al., 2018; Miles, Johnson, et al., 2018; Thatte et al., 2020). Accounting for these challenges is vital to our biodiversity hotspots, where heterogeneity is often at its highest and information on population genetic connectivity is crucial for management (Myers et al., 2000).

The Cape Floristic Region (CFR), found in the southwestern tip of South Africa with ~9000 plant species in only 90,000 km^2^ (Cowling et al., 1996; Goldblatt, 1997; Linder, 2003), 70% of which are endemic, is the most diverse extratropical area for plant biodiversity (Latimer et al., 2005; Linder, 2005; Rundel et al., 2016). The CFR is one of several Mediterranean Biomes, where high plant species diversity is typical (Cowling et al., 2015), yet the southwestern CFR alone is an outlier, with endemism equal to or higher than the neotropics (Latimer et al., 2005; Rundel et al., 2016). The high levels of endemism are associated mostly with fynbos (“fine bush”), which is the dominant and most speciose vegetation in the CFR (Cowling et al., 1992), covering half of the geographic area with >80% of its species (Goldblatt, 1978; Linder, 2003).

The CFR is a global and local conservation priority (Rouget et al., 2003), with its status as a UNESCO World Heritage Site due to its ancient cultural history, and evidence of some of the earliest development of symbolic human behavior that likely evolved as a result of the resource‐rich area (Marean, 2016; Marean et al., 2014). Today, the combination of a rich plant diversity and an equally rich cultural diversity in the CFR presents unique applications for biomedicine (Van Wyk, 2011). Unfortunately, human population growth in the CFR is the second highest among its Mediterranean Biome peers (Underwood et al., 2009), with agricultural and urban expansion devastating low‐lying coastal areas and threatening inland mountain refugia (Bomhard et al., 2005). By 2030, it is expected that 75% of the South African human population will live in urban areas (Cooperative Governance & Traditional Affairs, 2016); thus, conservation efforts within the CFR need to link anthropogenic activity with eco‐evolutionary dynamics (Alberti et al., 2020; Cilliers et al., 2014; Graham, 2017; Rebelo et al., 2011).

Anthropogenic impacts aside, CFR diversity has long been explained by a suite of environmental traits that interact on a landscape consisting of a highly heterogeneous array of microregions (Cowling et al., 2005; Latimer et al., 2005, 2006; Linder, 2003). Soil fertility varies from nutrient‐poor to mineral‐rich and has been hypothesized as a barrier among vegetation types on fine scales (Campbell, 1983; Linder, 2003). Mountain ranges, which create elevation gradients changing from sea‐level to over 2200 m within a few kilometers, have been identified as isolating barriers creating potential refugia (Verboom et al., 2009, 2015), and plant species richness has been positively correlated with altitude (Cowling & Lombard, 2002). Overall, CFR plant species diversity declines moving west to east (noted as “Levyn's Law”; Cowling et al., 2017) and has been associated with higher winter‐rainfall in the west to less rainfall seasonality in the east (Cowling et al., 1992, 1997, 1998; Cowling & Lombard, 2002).

In teasing apart what biotic and abiotic factors impact CFR diversity, there has been much debate on the evolutionary explanations. Some have suggested the high CFR diversity has been the result of historically high speciation rates, followed by re‐occurring extinction and colonization (Linder, 2005), a signature of instability within this heterogeneous landscape. Others have postulated that biome stability and topographic heterogeneity maintain species diversity (Colville et al., 2020; Cowling et al., 2005; Potts et al., 2015). Specifically, southwestern CFR biome stability created higher species diversity originating in the Pleistocene, with recent environmental instability explaining lower diversity in the eastern CFR (Cowling et al., 2005, 2017; Forest et al., 2018; Goldblatt, 1997; Manne et al., 2007; Proçhes et al., 2006). Other than studies of species richness, this latter hypothesis has seen support from fossil analyses (Cowling et al., 1999; Rector & Verrelli, 2010; Sauquet et al., 2009) and phylogenetics (Linder & Hardy, 2004; Pirie et al., 2016; Richardson et al., 2001; Valente et al., 2010). There has long been a call for microevolutionary analyses of gene flow and population structure in the CFR (Barraclough, 2006; Bergh et al., 2007; Cowling et al., 2009; Hardy, 2006; Latimer et al., 2006; Linder, 2006; Prunier & Holsinger, 2010), yet population genetic analyses that focus on demographic change for biogeographically diverse CFR plants are very rare (Lexer et al., 2014; Rymer et al., 2010). The few studies point to high phylogeographic diversity, but due to narrow sampling and an often focus on adaptive traits, neutral hypotheses of the evolutionary explanations and landscape drivers remain largely inconclusive (Lexer et al., 2013).

The endemic fynbos plant *Leucadendron salignum*, a shrub in the Proteaceae, is a suitable model for evaluating how neutral population processes are influenced by factors in the CFR (Cowling et al., 2005). Within the Proteaceae—the most prominent flowering fynbos group—the *Leucadendron* genus is considered basal, with its radiation potentially predating the origin of fynbos vegetation (Barker et al., 2004). *L. salignum* or “common sunshine conebush” is a uniquely biogeographically diverse CFR species (Barker et al., 2004), found across a range of soil types, rainfall regimes, and elevations, and inhabiting every area of fynbos. It is an evergreen diploid, dioecious plant, whose chloroplast (cp) DNA is maternally inherited (Pharmawati et al., 2004), with cpDNA and nuclear (nu) DNA reflecting seed and pollen dispersal, respectively. It is insect‐ and wind‐pollinated, with weakly developed serotiny (retention of seeds born in cones), and survives fire by resprouting from a lignotuber as well as serotiny for seed dispersal (Hattingh & Giliomee, 1989; Welsford et al., 2014, 2016). An historically abundant species, *L*.* salignum* has seen recent declines in lowland areas like other CFR species, whereas the lack of human interest in agriculture and development in montane fynbos retains large population sizes for the species in these areas (Rebelo et al., 2019).

Here, we sample *L*.* salignum* as a model to characterize molecular evolutionary and ecological patterns of CFR population genetic connectivity across spatial and temporal scales. This is the first study to use next‐generation DNA sequencing and broad‐ and fine‐scaled landscape genetic analyses to test overall hypotheses of CFR evolutionary stability, as well as effects of human land‐usage, in addressing several questions. First, do population genetic patterns mirror species‐level patterns of long‐term biome stability in the southwestern CFR (which predicts greater and older evolutionary genetic divergence in this region), in contrast to patterns of biome instability in the eastern CFR (which predicts more recent evolutionary divergence in this region)? Second, do CFR population genetic patterns also mirror species‐level patterns with evidence of local fine‐scale environmental factors, such as seasonal rainfall and elevation, shaping patterns of population genetic connectivity? Lastly, do we find evidence that anthropogenic activity, such as human land‐usage, has negatively impacted population genetic connectivity? Addressing these questions in the *L*.* salignum* model will help reveal how CFR population analyses shed light on CFR species diversity, as well as provide applied management plans with a means to identify where CFR genetic connectivity is most threatened.

## 2 MATERIALS AND METHODS

### 2.1 Study design and sampling

**FIGURE 1:**
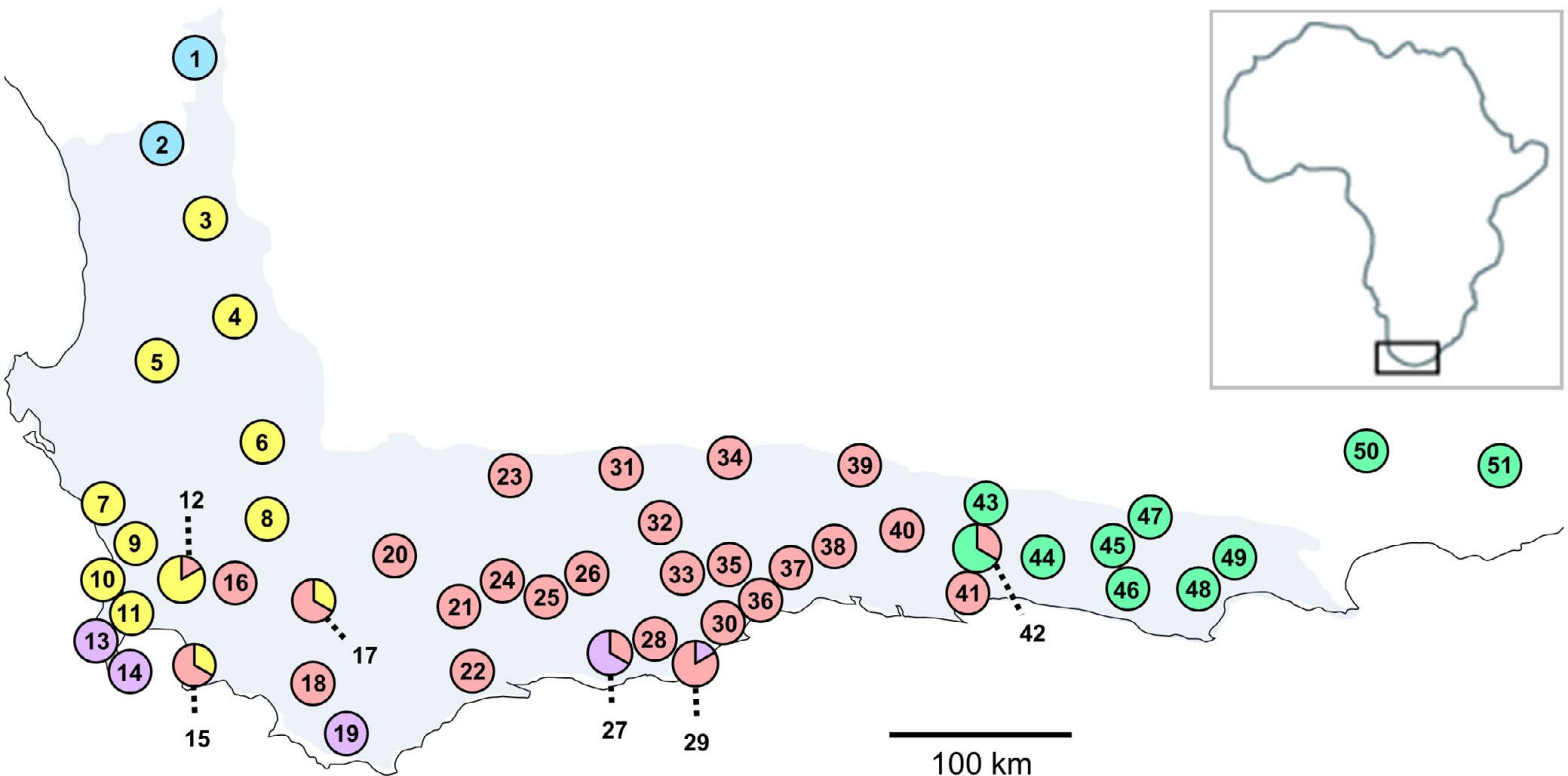
Map of southern South Africa with 51 sampled locales of 306 *Leucadendron salignum* (Table S1 for locale information). Pie charts provide geographic reference to color coding in phylogenetic (Figure 2), population structure (Figures 3 and 4), and population connectivity (Figure 5) analyses. Shaded area denotes recognized Cape Floristic Region (after Cowling et al., 2017)

We employed a constructed random sampling of 306 *L*.* salignum* individuals from 51 locales (each <0.01 km^2^) distributed across its biogeographical range (Figure 1; Table S1). Locales were identified from publicly available map data of vegetation types within fynbos habitat (SANBI, 2018), where *L*.* salignum* is found (Rebelo et al., 2019). We prioritized the sampling of CFR locales across climate regimes, elevation, vegetation types, and ecozones from dense mountain to coastal fynbos, as well as human land‐usage from agriculture to development (Figure S1).

We generated datasets of chloroplast (cp) and nuclear (nu) DNA to reflect the *L*.* salignum* dispersal of diploid embryos as seeds and haploid pollen, respectively. This approach is necessary to determine whether factors on the landscape impact gene flow differentially for seed and pollen dispersal (Holderegger & Giulio, 2010). For example, phylogenetic analyses of CFR plants that show higher pollen dispersal tend to evolve with lower seed dispersal (Tonnabel et al., 2014). Thus, as previously noted, pollen dispersal in *L*.* salignum* is facilitated by wind and insects (Hattingh & Giliomee, 1989; Welsford et al., 2014, 2016), and thus, it may be the case that while nuDNA (pollen‐related) patterns reflect high gene flow, that cpDNA (seed‐related) patterns are consistent with lower gene flow. With this multi‐tiered approach, the sampling of many locales was our focus for analyses of population connectivity to resolve spatial and environmental heterogeneity (Dyer, 2015; Dyer et al., 2010, 2012). In this respect, our cpDNA dataset sampled six individuals from each of the 51 locales, which results in 306 cpDNA alleles/sequences (i.e., 306 haploid samples), whereas our nuDNA dataset sampled four individuals from each locale, which results in 408 alleles/sequences (i.e., 204 diploid samples). This genetic sampling is the largest to date of any plant or animal in the CFR and is unusual in comparison to even other land‐usage population‐based studies outside this region (Miles et al., 2019).

### 2.2 Genetic marker collection

Sampled leaves were immediately placed in sealable bags of silica gel in the field and subsequently stored at room temperature to rapidly dry them for preservation. Genomic DNA was extracted using the DNeasy Plant Mini Kit (QIAGEN) according to the manufacturer's protocol. We designed a relatively simple and affordable approach that targeted hundreds of putatively neutrally evolving cpDNA and nuDNA fragments using degenerate PCR primers to generate SNP data (Data S1). We used the Nextera XT DNA sample preparation kit (Illumina, Inc.) and Illumina MiSeq (250 bp, paired end) next‐generation technologies to barcode and collect full sequence data. The resulting fasta files were aligned using the MUSCLE default parameters in the program Geneious v. 4.8.4 (Kearse et al., 2012), with postsequence filtering steps to enrich our dataset for single‐copy haploid and diploid fragments. Details of the data collection are presented in the Data S1.

### 2.3 Population structure and connectivity analyses

Unless noted otherwise, population genetic summary statistics and analyses were generated using the *PopGenome* v. 2.7.5 R package (Pfeifer et al., 2014). Estimates of per nucleotide site diversity (*θ*) were generated for each of the cpDNA and nuDNA datasets and for each locale using the average number of pairwise nucleotide differences (*θπ*) and the number of polymorphic sites (*θ*s). We estimated Tajima’s (1989) D for each of the cpDNA and nuDNA datasets to reflect the SNP frequency spectrum, which can provide insight into deviations from demographic equilibrium (e.g., population expansion). Estimates of genetic diversity between locales were calculated as pairwise *F*_ST_ values for each of the cpDNA and nuDNA datasets (using the estimator of Hudson et al., 1992). Following Hudson (2000), we used a permutation analysis with an in‐house script in R to test for deviations from panmixia (as in Miles, Dyer, et al., 2018). Specifically, this analysis pooled all locales, and randomly sampled allelic variation using our same sample sizes to reconstitute all locales, after which pairwise *F*_ST_ values were calculated. This simulation was repeated 1000 times and observed *F*_ST_ values were compared to these simulated distributions. These simulations determine the extent to which estimates of genetic diversity between locales are sensitive to the sample sizes of the number of individuals and the number of alleles for each locale for both the cpDNA and nuDNA datasets.

We performed a principal component analysis (PCA) on each of the cpDNA and nuDNA datasets to infer genetic clustering of individuals using the *gstudio* v. 1.5 R package (Dyer, 2016). We also used the Bayesian clustering program fastSTRUCTURE (Raj et al., 2014) with standard defaults to estimate the potential number of genetic clusters (*K*) for each of the cpDNA and nuDNA datasets. Samples were coded as individuals and run with 20 iterations for each *K* cluster. The program CLUMPAK (Kopelman et al., 2015) was used to summarize the output, which was visualized using DISTRUCT (Rosenberg, 2004). Both the PCA and fastSTRUCTURE analysis enable interpretation of genetic variation independent of the locales within which it was sampled, that is, locale‐labeling is assigned to individuals only after analysis is performed to identify unbiased clustering of genetic variation.

For analyses of genetic connectivity among locales, we employed a graph theoretical framework (Dyer et al., 2010) using the derived statistical measure of conditional genetic distance as cGD. The statistic cGD is estimated from the genetic covariance among all locales connected through a population network. Thus, cGD reflects the genetic connections among all locales in the network, in contrast to genetic distance estimators such as *F*_ST_ that make inferences about gene flow from pairwise dissimilarities only (Dyer et al., 2010). This genetic covariance measure as cGD was used to generate a population graph or “popgraph” for each of the cpDNA and nuDNA datasets, using the *popgraph* v. 1.5 R package (Dyer, 2017), where nodes represent sampled locales, and edges represent genetic connections among locales (as in Dyer et al., 2012).

Popgraph topologies can be used to depict parameters utilized in “social networks” to define characteristics of the population genetic network (as in Miles, Dyer, et al., 2018; Miles, Johnson, et al., 2018). This social network model evaluates genetic relationships among locales and relative contributions of key “actors” using mathematical graph theory (Wasserman & Faust, 1994). This model visually represents gene flow and “hubs” of connectivity among sampled locales. Social network node‐specific parameters including closeness, degree, betweenness, and eigenvector centrality were calculated using the *popgraph* v. 1.5 R package. Higher “closeness” values indicate higher genetic distance between a locale and other locales in the network, whereas lower “degree” values may indicate a locale is more relatively isolated from other locales in the network (Dyer, 2007). Higher “betweenness” values indicate a locale has relatively more paths that flow through it from other locales. “Centrality” reflects the extent to which a locale drives gene flow as a “hub” and reflects a sum of the other popgraph parameters noted above. Altogether, we interpret lower values of closeness and higher values of degree, betweenness, and centrality as indicative of areas in the popgraph with higher gene flow compared to the rest of the popgraph.

To identify statistical differences and congruence within and between popgraph topologies (e.g., whether patterns of connections and network parameters differ between cpDNA and nuDNA popgraphs), we employed a permutation analysis using the *gstudio* v. 1.5 R package (as in Dyer et al., 2012; Miles, Dyer, et al., 2018; Miles, Johnson, et al., 2018). Specifically, for each popgraph (cpDNA and nuDNA, respectively), each node (representing each locale) was fixed on the popgraph (constructed as noted above), while we randomly permuted the edges that connect the locale nodes. For each social network node‐specific parameter that we estimated (e.g., “closeness”), we simulated 1000 randomized popgraphs, each drawn from a distribution that included the observed parameter estimates within that popgraph. For each test, statistical significance was assessed by determining the probability of each observed network parameter value given our simulated popgraphs generated from each node‐specific parameter.

### 2.4 Landscape and genetic association analyses

We used several approaches to interpret the relationship between genetic and landscape variation among our 51 *L*.* salignum* locales. Isolation‐by‐distance models predict that as geographic distance between populations increases, so does genetic differentiation between populations (Slatkin, 1993), whereas general isolation‐by‐resistance models (McRae, 2006) predict that features on the landscape, including geographic distance, provide “resistance,” either by reducing or even facilitating gene flow (Miles et al., 2019). Thus, through these association analyses, we may interpret a CFR landscape variable as contributing to overall patterns of gene flow in *L*. *salignum*, and with further investigation of those patterns, we can then identify where those patterns increase or decrease population connectivity. One assumption in these analyses is that contemporary landscape measures reflect evolutionary and ecological measures, especially for very recent events, such as those involving anthropogenic activities (Leigh et al., 2019; Miles et al., 2019). Another consideration is that measures of landscape variables also include variance across time (Anderson et al., 2010). In our case here, variables on elevation, precipitation, and vegetation have been long‐recognized as evolutionary stable processes (Cowling et al., 2017), and so we may expect these variables have more power to explain relatively older evolutionary genetic events. While human land‐usage has been relatively recent, previous analyses also show that it is possible to detect changes in the CFR that reflect contemporary disturbance (Slingsby et al., 2020).

Our first approach used a generalized linear mixed model (GLMM), which accounts for nonindependence of distance matrices (Clarke et al., 2002), and has been shown to be a powerful approach to test multiple hypotheses and investigate nonindependent features in landscape genetic analyses (Row et al., 2017). We used the function lme of the *nlme* R package (Pinheiro et al., 2013) and used the Akaike information criteria (AIC) to inform the best models. In this analysis, the pairwise *F*_ST_ values from the cpDNA and nuDNA datasets were used as our proxies for genetic distances between all locales and constituted our single dependent variable in the GLMM. We employed four landscape features as independent variables (Figure S1) that are commonly used to model patterns of CFR plant species richness in South African biomes (Thuiller et al., 2006). These four variables are as follows: (i) elevation (ELE), via WorldClim data (value expressed in meters); (ii) annual rainfall (PPT), via WorldClim data (value expressed in millimeters); (iii) seasonal rainfall concentration (CON), ratio of winter to summer seasonal values (see Schulze, 1997); and (iv) vegetation type (VEG), reflecting different vegetation substrates, soil composition, and fertility as categorized by the SANBI (2018) National Vegetation Map study (resolution of 1 km^2^ regions over a 1000 × 2000 grid). As we have previously hypothesized, land‐usage change may have the potential to fragment population connectivity. Thus, we added a fifth landscape feature as an independent variable in the GLMM to test hypotheses about the potential impact of land‐usage (LAN). We used the GeoTerraImage (2017) Southern Africa land‐cover dataset (30 × 30 m raster cell resolution), which reflects both natural and human‐altered landscape types, the latter of which includes plantations and cultivated agriculture, as well as urban and rural settlement (Figure S1).

We acquired raster image files for each of the five independent landscape variables, as noted above, and cropped them to fit the geographic area of the 51 sampled locales (Figures 1 and S1). Because spatial and landscape resistance distances are inherently correlated, for example, as one increases in elevation between two locales, there is also an increase in geographic distance, we included geographic distance as a covariate within the GLMM to assess the relative independent contributions of each landscape variable. Geographic distances (GEO) between locales were estimated from latitude and longitude coordinates as Euclidean distances using the PASSaGE program (Rosenberg & Anderson, 2011). For each of the five landscape variables, the mean and variance of the raster pixel values among all pairwise connections of the 51 locales were each calculated using the *raster* R package (Hijmans & van Etten, 2012). The mean and variance calculated between all locales reflect different spatial properties. For example, the mean of an increase, followed by a decrease, and again an increase in elevation between two locales could be identical to a mean score that reflects “no change” in elevation between two locales. In this way, we can capture this variation in change between two points, which is what potentially influences gene flow between two points on the landscape. In addition, calculating landscape resistance distance as the difference between two locale points misses critical information. That is, elevation at locale 1 and elevation at locale 2 is identical and thus the difference between these two locale points is “0,” but dramatic changes in elevation occur between those two points, as we observe over even short geographic distances in the CFR. These mean and variance statistics were used as landscape resistance measures in separate GLMM analyses of each of the cpDNA and nuDNA datasets to test for relationships between landscape and genetic variables.

Our second approach used a permutation analysis with the *gstudio* v. 1.5 R package (as above) to identify significant relationships between genetic connectivity (estimated as cGD), and our landscape variables, as in previous analyses (Dyer et al., 2012; Miles, Dyer, et al., 2018; Miles, Johnson, et al., 2018). Specifically, for each of the cpDNA and nuDNA datasets, each of the 51 sample locales (or nodes) was fixed on the landscape of the popgraph, which was estimated using cGD, or the genetic covariance among all locales (as above). As above, nodes of the popgraph reflect the locales and connections reflect the cGD measures or edges of the popgraph. We overlaid the connections of the popgraph on each of the raster maps of each of the five landscape variables (ELE, PPT, CON, VEG, LAN) and then calculated the observed mean and variance resistance distances of all the pairwise connections that separate the locales. We then simulated 1000 randomized popgraphs that were constrained by edge density but also by the edge distribution on individual nodes, generating a null distribution of popgraphs. These null distributions for each landscape feature generated from simulations (using the observed popgraph data) were then compared to the observed popgraph for each landscape variable to look for differences in congruence. Statistical significance was assessed by determining the probability of the observed values given our simulated popgraphs of each landscape variable. This analysis randomizes all the genetic, landscape, and spatial variables observed among all locales to simply ask whether specific landscape features are more often associated with genetic distances between locales (i.e., genetic connectivity or gene flow), than expected by chance.

### 2.5 Estimates of molecular evolutionary relationships

We conducted several analyses to place genetic divergence among locales and lineages in an evolutionary framework. While we are interested in the phylogenetic structure among locales and lineages, we are also interested in generating date estimates for this genetic structure. Although prior phylogenetic tree analyses of the *Leucadendron* genus have been published (Barker et al., 2004; Hoffmann et al., 2015; Tonnabel et al., 2014), molecular clock divergence estimates have not been previously estimated for this group. Thus, we generated molecular clock divergence estimates for *Leucadendron* using previously published species nucleotide data (Table S2) and the approach of Valente et al. (2010), which estimated divergence times of the closely related South African *Protea* genus using a relaxed Bayesian MCMC approach implemented in BEAST (Drummond & Rambaut, 2007). We also collected nucleotide sequence data for a closely related outgroup (*Leucadendron laureolum*) to align with our *L*.* salignum* data generated in this study. With this outgroup and the approach of Valente et al. (2010), we generated a rooted *L*.* salignum* phylogenetic tree with date estimates for interpreting geographic and evolutionary diversity. Details of the phylogenetic analyses and dating estimates are presented in the Data S2.

## 3 RESULTS

After multiple levels of postdata filtering, the resulting dataset from our sampling of 51 *L*.* salignum* locales included >100 SNPs from 306 haploid cpDNA sequences and >800 SNPs from 408 nuDNA sequences. Statistics reflecting SNP frequency spectra for marker datasets (cpDNA, *D* = −0.71; nuDNA, *D* = −1.60) are not significant and show no deviation from demographic equilibrium. Nucleotide sequence diversity for the overall dataset is higher for nuDNA than it is for cpDNA; however, estimates within locales are considerably lower, especially for cpDNA (Table S3), suggesting significant genetic variation is distributed between locales. This is indeed the case as we find significant genetic divergence among many locales (Figure S2), and much higher overall structure in cpDNA (*F*_ST_ = 0.81) compared to nuDNA (*F*_ST_ = 0.10).

**FIGURE 2:**
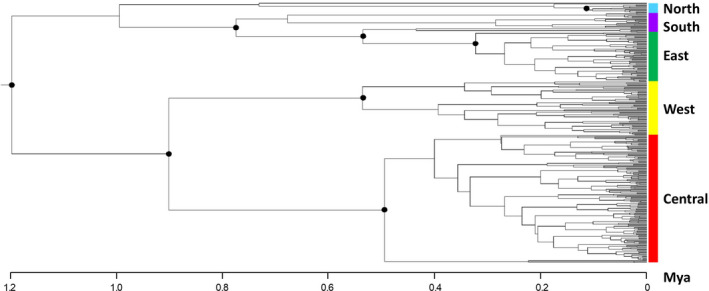
Consensus BEAST phylogenetic tree topology of 306 *Leucadendron salignum* chloroplast DNA SNP haplotypes, rooted by outgroup, with estimates of genetic divergence times (see Materials and Methods). Black dots on nodes represent major branches with support >75%

Several key observations emerge from the BEAST analysis of cpDNA (Figure 2), which support evolutionary groups that mirror geographic clustering (Figure 1). We find support for four groups in the western CFR (labeled here as “north,” “west,” “south,” “central”), whereas there is support for a single group in the eastern CFR (labeled as “east”). The estimated coalescence for the population sample at 1.17 Mya (95% CI = 0.73–1.63 Mya) is followed by two highly supported groups, one that includes the west and central groups, and one that include the north, south, and east groups. The west, central, and south groups each have estimated coalescent times coinciding at ~0.50 Mya (0.26–0.78), with the east group emerging more recently from an apparent common ancestor in the southwestern CFR at ~0.31 Mya (0.17–0.47), and the smaller north group emerging more recently at ~0.17 Mya (0.05–0.30). The overall estimate of coalescence of the nuDNA lineages at ~1.7 Mya was consistent with that of the cpDNA tree. However, unlike the cpDNA tree, there was no significant internal node support for any individual nuDNA groups. To independently confirm these BEAST results, we performed a simple model‐free neighbor‐joining (NJ) tree analysis using MEGA (Kumar et al., 2016), which supported the BEAST topologies of the cpDNA lineages as well as the nuDNA polytomy.

**FIGURE 3:**
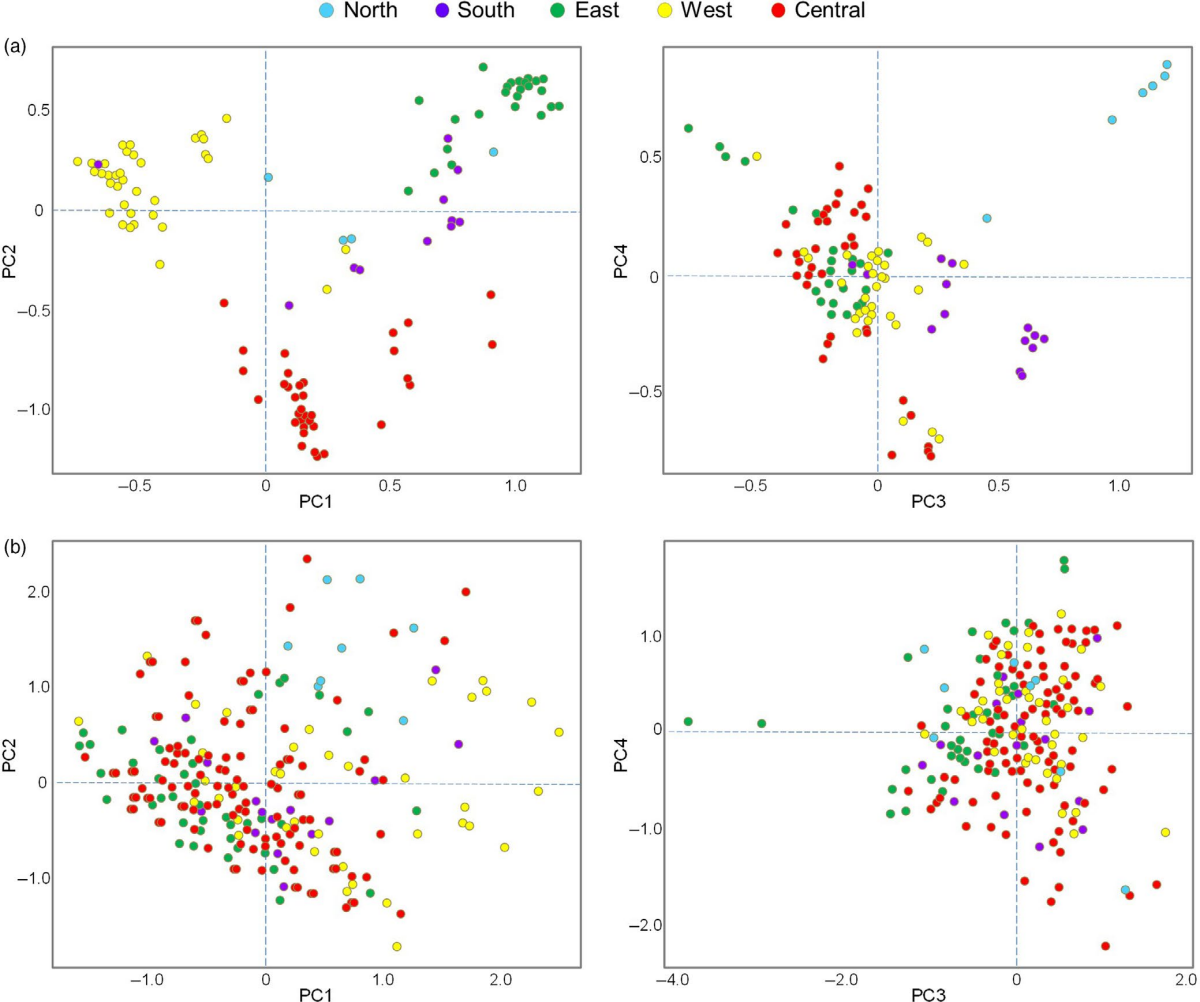
Bi‐plots of the first four principal components of *Leucadendron salignum* datasets using (a) chloroplast DNA SNPs from 306 sequences, and (b) nuclear DNA SNPs from 408 sequences. Color‐coding reflects the five major phylogenetic tree clusters (Figure 2)

**FIGURE 4:**
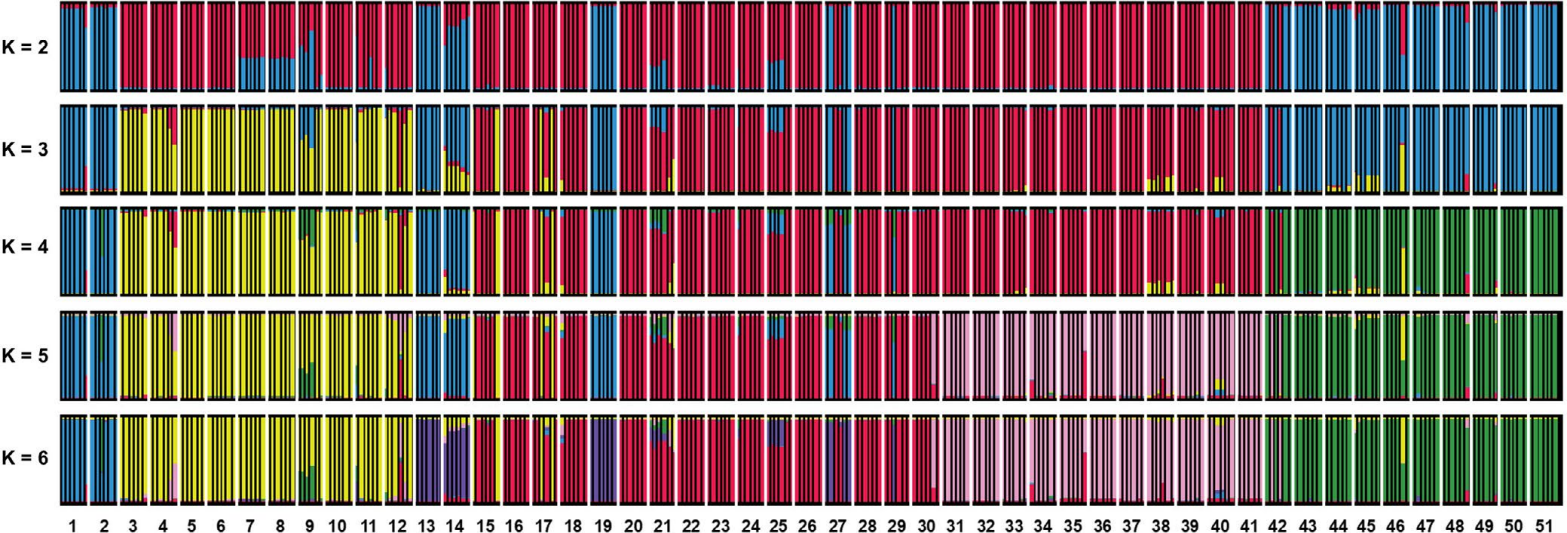
fastSTRUCTURE analysis of 306 *Leucadendron salignum* chloroplast DNA SNP haplotypes from 51 sampled locales (see Figure 1 for geographic sampling, Table S1 for locale information)

The PCAs of our cpDNA and nuDNA datasets also exhibit contrasting patterns with cpDNA reflecting geographic clustering, whereas nuDNA exhibits little separation (Figures 1 and 3). The first and second PCs of cpDNA separate west, central, and east groups (PC1 = 29.1%, PC2 = 19.0%), with the third and fourth PCs separating south and north groups (PC3 = 7.1%, PC4 = 6.4%). The nuDNA PCA reflects very little variance explained (total PC1–4 = 20.3%). The fastSTRUCTURE analyses mirror the PCA results. The cpDNA analysis for *K* = 2–6 (additional *K* runs resulted in no further clustering) reflects multiple clusters moving geographically west to east (Figures 1 and 4). The only exception is the south group (*K* = 6), which includes individuals that are geographically distantly separated, but have in common their southwestern coastal locations. Notably, the central group additionally separates into multiple geographic clusters (*K* = 5), and coincidentally, the cpDNA PCA identified this more subtle genetic separation within the central region (PC5–6 = 8.2%). On the other hand, the nuDNA fastSTRUCTURE analysis found no genetic clustering (i.e., could not reject a model of *K* = 1, Figure S3).

**FIGURE 5:**
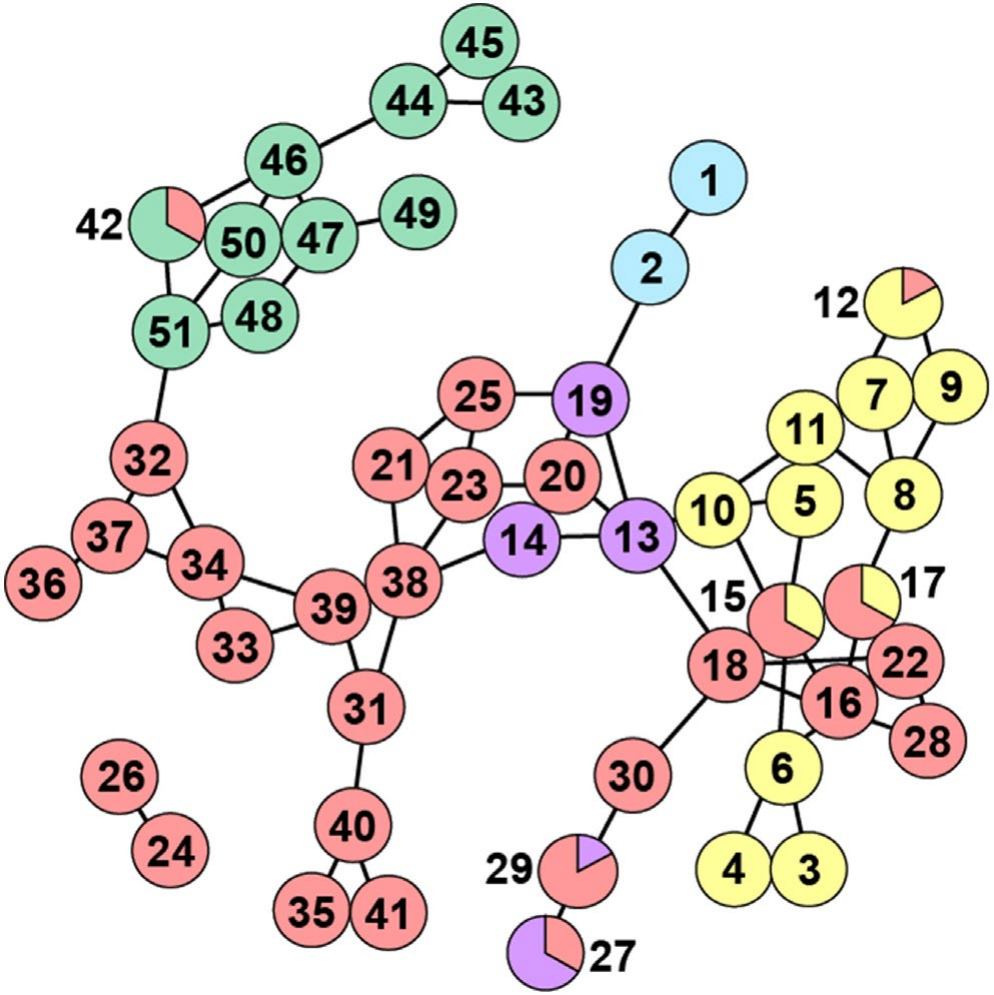
Popgraph of genetic connectivity among 51 sampled locales of *Leucadendron salignum* chloroplast DNA (see Figure 1 for geographic sampling, Table S1 for locale information). Color‐coding reflects the five major phylogenetic tree clusters (Figure 2)

In our analysis of the network parameters from the cpDNA popgraph, we found that connections among eastern CFR locales have significantly higher connectivity (i.e., lower “centrality,” *p* = 0.0002) and are more genetically similar (i.e., lower “closeness,” *p* = 0.00009) in contrast to connections anywhere else in the popgraph (Figure 5; Table S4). The measure of “betweenness” reflects nodes that significantly influence connectivity across the network as a whole. In the cpDNA popgraph, the locales with the highest “betweenness” values are those found at each of higher elevation, within the Cape Peninsula, and also along the southwestern coast (Table S4 and Figure S4). When locales are disconnected from the popgraph network it does not reflect “isolation” but instead signifies that they do not contribute added information to patterns of genetic connectivity among the other locales. This was the case for only two locales in the cpDNA popgraph (Figure 5). In contrast, the locales of the nuDNA popgraph are almost entirely disconnected; that is, locales equally share nuDNA allelic variation, indicating no distinct pattern of gene flow (Figure S5). This panmictic population genetic pattern for nuDNA is consistent with the BEAST, PCA, and fastSTRUCTURE results.

The landscape genetic analysis performed in the GLMM shows that geographic distance explains a significant proportion of genetic differentiation (measured as *F*_ST_) in both cpDNA and nuDNA datasets (9% and 15%, respectively), but that landscape variables explain very little variance (Table S5). In contrast, our simulation analyses that assessed the relationship between landscape features and genetic connectivity (measured as cGD) provided significant results (Figure S6). These results are consistent with the cGD measure as a statistically more powerful proxy of gene flow as it reflects shared variation through the entire network, whereas *F*_ST_ reflects dissimilarity, and not explicitly connectivity among locales (Dyer et al., 2010). As noted previously, the nuDNA data show no patterns of genetic structure or connectivity; whereas, landscape permutation analyses with the cpDNA popgraph reveal that each of ELE, PPT, CON, and LAN variables were significant (Figure S6). These analyses find that observed genetic connections on the popgraph occur significantly more often in areas with (i) lower elevations, (ii) higher annual precipitation, and (iii) higher winter‐rainfall concentration than predicted by the simulations. Further investigation into the significant LAN results found that compared to the simulations (Figure S6), genetic connections on the popgraph were significantly underrepresented in areas where “fynbos thicket” (i.e., a mosaic of Cape fynbos and subtropical thicket, Vlok et al., 2003) and “commercial cultivation” (i.e., commercial agriculture) occur (Figure S1). Thus, these two land‐usage types appear to significantly reduce genetic connectivity among locales where these features exist. As previously noted, a GLMM is a valuable approach as it models the potential nonindependence of landscape features; nonetheless, the four landscape variables that showed significant relationships with genetic connectivity are only weakly correlated with each other (*r* ^2^ [ELE × PPT] = 0.37; *r* ^2^ [ELE × CON] = 0.05; *r* ^2^ [ELE × LAN] =0.05; *r* ^2^ [PPT × CON] = 0.22; *r* ^2^ [PPT × LAN] = 0.47; *r* ^2^ [CON × LAN] = 0.0002).

## 4 DISCUSSION

Using the CFR‐wide *L*.* salignum* plant as a population and landscape genetic model, we set out to test hypotheses of the evolutionary and environmental factors that drive the rich CFR plant species diversity. Our analyses find that while pollen‐related genetic signatures reflect a panmictic population with gene flow largely uninhibited, seed‐related genetic signatures reflect high population genetic structure, and demographic patterns of relatively deeper and older genealogies in the southwestern CFR compared to lineages in the northern and eastern CFR. Although we find several environmental factors associated with population genetic connectivity, a striking result was the association of human land‐usage with reduced gene flow. We discuss how these patterns are consistent with evolutionary hypotheses, the implications for management strategies, and future directions to evaluate CFR genetic models.

### 4.1 Contrasting dispersal patterns of genetic differentiation

Our results from nuclear and chloroplast genetic markers are a sharp reminder of how sampling only one genetic mode of inheritance could potentially provide an incomplete picture. Although *L*.* salignum* nuDNA shows higher genetic diversity compared to cpDNA (as is expected for biparental, recombining nuDNA markers; Wolfe et al., 1987), in contrast to cpDNA, nuDNA shows very little population structure, genetic connectivity, or phylogeographic resolution. Patterns of cpDNA variation reflect the hypothesis of historically consistent CFR edaphic and climatic conditions (Cowling et al., 2017; Potts et al., 2015), which likely selects for lower seed dispersal in habitat‐specialist taxa (Lopez et al., 2008; Olivieri et al., 2015). In contrast, patterns of pollen dispersal in *L*.* salignum* unsurprisingly reflect pandemism, consistent with phylogenetic analyses of CFR plants that show higher pollen dispersal tends to evolve with lower seed dispersal (Tonnabel et al., 2014). While pollen dispersal in *L*.* salignum* is facilitated by wind and insects (Hattingh & Giliomee, 1989; Welsford et al., 2014, 2016), the lack of pollen geographic variation here does not suggest that pollinator movement in the CFR is also unaffected by environmental factors. Given the different pollen and seed patterns here, population genetic analyses of CFR pollinators could elucidate the co‐evolutionary relationship they have with plant diversity (Eimanifar et al., 2018).

### 4.2 Signatures of broad‐ and fine‐scale patterns of CFR genetic diversity

Population genetic analyses for *L*.* salignum* reveal contrasting patterns of CFR diversity across different spatial and temporal scales. Population genetic structure overall is significantly high, yet much of this variation is consistently found between southwestern CFR locales in very close geographic proximity. In fact, geographic distance explains a significant, yet small, overall proportion of population genetic structure on broad‐scales (9% of cpDNA, and 15% of nuDNA diversity, Table S5). As previously noted (e.g., Cowling et al., 2017; Forest et al., 2018), and predicted here, phylogeographic evolutionary analyses show lineages from the southwestern CFR exhibit higher genetic structure and older estimated ages than lineages found across the most distantly geographically separated locales in the CFR. That is, a simple isolation‐by‐distance model cannot explain patterns of genetic divergence observed here for either marker type. Interestingly, although eastern lineages appear to have a derived ancestry from the west, their estimated ages are not necessarily recent. That is, eastern lineages appear to have a single evolutionary root and very low population structure, but our coalescent analyses find an estimated age of ~500 Kya, and no evidence of reduced nucleotide diversity, inconsistent with a very recent colonization event (Linder & Hardy, 2004). These overall geographic patterns are consistent with higher phylogenetic diversity, but older divergence of Cape clades in the west, compared to lower phylogenetic diversity and more recent species radiation in the east (Richardson et al., 2001; Schnitzler et al., 2011; Valente et al., 2010; Verboom et al., 2009).

While the few previous CFR plant phylogeographic studies were limited in sample size and scope, they revealed consistently high population structure (Lexer et al., 2013, 2014). Here, population genetic signatures are consistent with the hypothesis that southwestern CFR high environmental and evolutionary diversity is the result of Pleistocene climate stability, whereas eastern CFR diversity is the result of climate instability during the same period (Cowling et al., 2015; Huntley et al., 2016; Linder, 2008; Verboom et al., 2009)—essentially reflecting “two CFRs” (Cowling et al., 2017). This theory predicts that biome stability in the southwestern CFR would foster a deep, evolutionarily stable *L*.* salignum* genealogical diversity, but with reduced gene flow and continuous lineage divergence, without extinction, over even short geographic distances due to a highly heterogeneous landscape. While biome diversity in the east is exceptionally rich (Cowling & Potts, 2015), its instability has evolved a *L*.* salignum* genetic diversity with signs of constant bottlenecks (which erode genealogical diversity), and evidence of invasion from older southwestern lineages. This latter explanation comes from the observation in our phylogenetic analysis that eastern lineages have a single common ancestor (derived from southwestern lineages) with reduced phylogenetic diversity. The southwestern CFR may act as an evolutionary genetic “source,” typified by genetic structure that is higher and older, that feeds into the eastern and northern CFR, which may appear as evolutionary genetic “sinks” typified by re‐occurring lineage extinction and colonization from the southwest.

### 4.3 Genetic connectivity in the CFR is shaped by biotic and abiotic factors

Landscape genetic results show that genetic connectivity among geographically distant locales is significantly associated with lower elevation and higher winter‐rainfall. This overall pattern mirrors previous studies showing declines in species richness moving west to east that are consistently associated with lower winter‐rainfall (Cowling et al., 2017). By marrying our patterns of gene flow with patterns of topographic heterogeneity (Figures 1 and 5; Table S4 and Figure S4), we can identify geographic areas with conservation implications. Our social network analyses find the greatest drivers of gene flow are geographically distant locales in a stretch of lowland elevation along the southwestern coast, for example, Cape Peninsula, across False Bay, and the Cape Agulhas Plain, into the eastern CFR. Interestingly, popgraph results show that this southern coastal route also maintains connectivity among clusters of southwestern locales separated along low elevation routes moving in two directions. For example (Figures 1 and S4), clusters are separated by the Cederberg, Boland, and Hottentot and Holland mountain ranges that run north to south, with smaller pockets separated by the Hex River and Riviersonderend ranges. Running west to east we see clusters separated by the Cape Fold Mountains into the Langeberg and Swartberg ranges. Although locales in these ranges are distantly separated, they maintain high genetic connectivity running west to east linked by lowland elevation, whereas moving from coastal to montane locales, we see low genetic connectivity as a result of the parallel mountain ranges acting as barriers. These high‐resolution genetic patterns strongly coincide with species richness patterns predicted by large studies of biome stability and topographic heterogeneity at the global scale as well as within the CFR (Colville et al., 2020).

Certain CFR environmental features, such as elevation and precipitation, may be biogeographically correlated, along with vegetation type; thus, our statistical model was designed to account for these associations to identify factors that may play independent roles in shaping population structure. For example, the observation that fynbos thicket is associated with reduced gene flow in the east is also coincident with seasonal winter‐rainfall showing a similar pattern; however, these variables are not always naturally associated (Becker et al., 2015; Potts et al., 2013). The subtropical thicket of southern Africa was hypothesized to have undergone great declines during colder glacial periods of the Pleistocene (Cowling et al., 2005; Vlok et al., 2003), which as noted previously, may have resulted in rapid species turnover and extreme bottlenecks in the east. This consistent upheaval may have had an effect on the evolutionary dynamics of population connectivity of flora associated with subtropical thicket (Potts et al., 2013). In fact, our exhaustive search identified only a few eastern pockets of *L*.* salignum* (Figure 1) nestled within dense thicket, and indeed, these eastern *L*.* salignum* lineages exhibit more recent, late Pleistocene coalescence. Our population genetic analyses suggest that the consistent evolutionary turnover of eastern CFR types, such as subtropical thicket, may have potentially both depressed invasion of and increased fragmentation of eastern fynbos lineages, such as *L*.* salignum*.

### 4.4 The impact of human activity on CFR diversity

Our project was initiated by the need to document how anthropogenic activity impacts biodiversity hotspots, like the CFR, through the use of evolutionary genetics. Thus, the observed genetic signature of negative anthropogenic impact is of interest. Environmental factors, such as elevation and precipitation, appear to have long‐impacted *L*.* salignum* genetic diversity, dating to at least the Pleistocene; however, human land‐usage is expected to impact the CFR in only the last ~300 years (Gaertner et al., 2016; Rebelo et al., 2011). Recent studies show that anthropogenic effects on allele frequencies and gene flow may take a considerable number of generations, depending on effective population sizes and standing genetic diversity, unless these effects are severe (Johnson & Munshi‐South, 2017; Leigh et al., 2019; Miles et al., 2019). Thus, our result with human land‐usage underlies the severe impact that anthropogenic activity may have on gene flow, especially in coastal and agricultural areas of the southwestern CFR, where development has exponentially expanded (Horn & Van Eeden, 2018). This development into coastal lowlands includes not only areas most vital to maintaining gene flow among the most geographically separated CFR locales, but also where we identify some of the most ancestral, oldest, and highest levels of genetic structure in the CFR overall.

This anthropogenic signature is of great concern given that 75% of South Africa's human population is expected to live in urban areas by 2030 (Cooperative Governance & Traditional Affairs, 2016). As anthropogenic activity fragments the landscape and reduces gene flow, we artificially shift the adaptive landscape (Wright, 1982) and select for very different life‐history traits that inhabit narrow geographic areas. Urban evolutionary studies have shown that in selecting for potential urban “ecotypes” (Johnson & Munshi‐South, 2017; Miles, Dyer, et al., 2018; Miles, Johnson, et al., 2018; Rost et al., 2009; Winchell et al., 2016), we stand to bottleneck vital CFR genotypic and phenotypic variation that has accumulated over millions of years of evolution. This study reminds us that while there is a need to focus on rapidly growing urban areas, we must be cognizant of other land‐usage such as agriculture as it also erodes vital biodiversity.

### 4.5 Future directions and applications

In identifying both evolutionary and environmental features associated with population connectivity in the CFR, several future directions emerge. First, *L*.* salignum* is an appropriate biogeographic and neutral model to generate hypotheses, but we need replication from other biogeographically diverse taxa for comparison, and not for plants alone. Lexer et al., (2013) noted that the heterogeneous and complex mosaic landscape is similar for both plants and animals alike, and examining diverse taxa can identify how these drivers are similar across phylogenetic time, mating systems, and genomes. The limited number of animal studies to date in the CFR shows far less phylogeographic diversity and evidence of range expansion consistent with the impact of Pleistocene climatic changes (Daniels et al., 2007; Portik et al., 2011; Tolley et al., 2006). Yet, this result is taken with caution given the limited sampling design, as well as the fact that some studies focus on animals of conservation interest but with current ranges and population sizes that deviate greatly from historical ones (Smit et al., 2007; Van Hooft et al., 2002). Thus, using larger and more sampling locales to disentangle the complex demographic histories of these taxa will be hard‐pressed to reveal the underlying drivers of CFR diversity and species divergence. In this respect, studies of unthreatened species with low conservation priority, but which have broad distributions such as the current study, have great conservation implications as they can help build neutral evolutionary models with which to test patterns from species of management concern.

A related challenge is teasing apart the relationship between geographic range and population genetic diversity, especially for neutral traits (McDonald et al., 1996; Morueta‐Holme et al., 2013; Slingsby & Verboom, 2006). As environmental history is an important factor in driving CFR evolutionary change, then sampling southwestern and eastern species with similar geographic ranges may show similarly high diversity/older lineages and low diversity/recent lineages, respectively. In addition, comparative phylogeographic analyses among Mediterranean biomes with similarly high diversity (Feliner, 2014) can reveal drivers unique to the CFR. That is, we may predict that signatures of genetic diversity and phylogeographic structure are similar across these biomes, but that landscape genetic analyses identify unique drivers and thus unique conservation priorities. Even within the CFR, we find that no one landscape feature alone can explain the phylogeographic diversity. Lower elevation and vegetation types alter connectivity in different ways, and thus, an intense sampling along specific mountain ranges, substrates, and watersheds across the CFR is needed to separate their influence on fine scales. One example here is from the association that “thicket” has with lower *L*.* salignum* gene flow in the eastern CFR. Potts (2017) conducted phylogeographic analyses of Albany subtropical thicket on fine scales and showed that watersheds have been long‐term barriers to gene flow creating isolated catchments that reflect evolutionary conservation units. By fine‐scale sampling within the CFR, we may reveal that these catchments isolate *L*.* salignum* populations as well. These similar patterns across species lend support to these local ecosystems needing consideration as evolutionary conservation units.

While our results shed further light on long‐term environmental features to aid in conservation priorities (such as lowland and coastal fynbos), other CFR species may reveal other priorities. For example, our study supports the need of fine‐scaled population network analyses that target contemporary factors of high threat such as human impact. We need a multi‐tiered approach to understanding CFR biome evolution (Potts et al., 2015) that includes CFR population genetic studies to create a narrative of the consistent eco‐evolutionary factors driving long‐ and short‐term diversity. As the affordability of large‐scale genetic data collection continues, our approach can be applied to any CFR species to help guide conservation plans.

## CONFLICT OF INTEREST

The authors declare no competing interests with research described in this paper.

## Supporting information

Supplementary Material

## Data Availability

Data for this study are available at the Dryad Digital Repository: https://doi.org/10.5061/dryad.612jm642glink.

## References

[eva13185-bib-0001] Alberti, M. (2015). Eco‐evolutionary dynamics in an urbanizing planet. Trends in Ecology and Evolution, 30, 114–126. 25498964 10.1016/j.tree.2014.11.007

[eva13185-bib-0002] Alberti, M. , Palkovacs, E. P. , Des Roches, S. , De Meester, L. , Brans, K. I. , Govaert, L. , Grimm, N. B. , Harris, N. C. , Hendry, A. P. , Schell, C. J. , Szulkin, M. , Munshi‐South, J. , Urban, M. C. , & Verrelli, B. C. (2020). The complexity of urban eco‐evolutionary dynamics. BioScience, 70, 772–793.

[eva13185-bib-0003] Anderson, C. D. , Epperson, B. K. , Fortin, M. J. , Holderegger, R. , James, P. , Rosenberg, M. S. , Scribner, K. T. , & Spear, S. (2010). Considering spatial and temporal scale in landscape‐genetic studies of gene flow. Molecular Ecology, 19, 3565–3575. 20723051 10.1111/j.1365-294X.2010.04757.x

[eva13185-bib-0004] Auffret, A. G. , Kimberley, A. , Plue, J. , & Walden, E. (2018). Super‐regional land‐use change and effects on the grassland specialist flora. Nature Communications, 9, 3464. 10.1038/s41467-018-05991-y PMC6110833 30150739

[eva13185-bib-0005] Ballare, K. M. , & Jha, S. (2020). Genetic structure across urban and agricultural landscapes reveals evidence of resource specialization and philopatry in the Eastern carpenter bee, *Xylocopa virginica* . Evolutionary Applications. 10.1111/eva.13078 PMC7819568 33519961

[eva13185-bib-0006] Balmford, A. , & Bond, W. (2005). Trends in the state of nature and their implications for human well‐being. Ecology Letters, 8, 1218–1234. 21352446 10.1111/j.1461-0248.2005.00814.x

[eva13185-bib-0007] Barker, N. P. , Vanderpoorten, A. , Morton, C. M. , & Rourke, J. P. (2004). Phylogeny, biogeography, and the evolution of life‐history traits in Leucadendron (Proteaceae). Molecular Phylogenetics and Evolution, 33, 845–860. 15522808 10.1016/j.ympev.2004.07.007

[eva13185-bib-0008] Barraclough, T. G. (2006). What can phylogenetics tell us about speciation in the CFR. Diversity and Distributions, 12, 21–26.

[eva13185-bib-0009] Becker, C. H. , Coetsee, C. , Cowling, R. M. , & Potts, A. J. (2015). The local landscape boundary between the Albany subtropical thicket and Nama‐Karoo shrubland is not influenced by edaphic factors. South African Journal of Botany, 101, 107–111.

[eva13185-bib-0010] Bergh, N. G. , Hedderson, T. A. , Linder, H. P. , & Bond, W. J. (2007). Palaeoclimate‐induced range shifts may explain current patterns of spatial genetic variation in renosterbos. Taxon, 56, 393–408.

[eva13185-bib-0011] Bomhard, B. , Richardson, D. M. , Donaldson, J. S. , Hughes, G. O. , Midgley, G. F. , Raimondo, D. C. , Rebelo, A. G. , Rouget, M. , & Thuiller, W. (2005). Potential impacts of future land use and climate change on the Red List status of the Proteaceae in the Cape Floristic Region, South Africa. Global Change Biology, 11, 1452–1468.

[eva13185-bib-0012] Campbell, B. M. (1983). Montane plant environments in the Fynbos Biome. Bothalia, 14, 283–298.

[eva13185-bib-0013] Centeno‐Cuadros, A. , Hulva, P. , Romportl, D. , Santoro, S. , Stribna, T. , Shohami, D. , Evin, A. , Tsoar, A. , Benda, P. , Horacek, I. , & Nathan, R. (2017). Habitat use, but not gene flow, is influenced by human activities in two ecotypes of Egyptian fruit bat (*Rousettus aegyptiacus*). Molecular Ecology, 26, 6224–6237. 28950408 10.1111/mec.14365

[eva13185-bib-0014] Charlesworth, D. , & Charlesworth, B. (1987). Inbreeding depression and its evolutionary consequences. Annual Review of Ecology and Systematics, 18(1), 237–268.

[eva13185-bib-0015] Cilliers, S. , du Toit, M. , Cilliers, J. , Drewes, E. , & Retief, F. (2014). Sustainable urban landscapes: South African perspectives on transdisciplinary possibilities. Landscape and Urban Planning, 125, 260–270.

[eva13185-bib-0016] Clarke, R. T. , Rothery, P. , & Raybould, A. F. (2002). Confidence limits for regression relationships between distance matrices: Estimating gene flow with distance. Journal of Agricultural Biological and Environmental Statistics, 7, 361–372.

[eva13185-bib-0017] Colville, J. F. , Bealec, C. M. , Forest, F. , Altwegg, R. , Huntley, B. , & Cowling, R. M. (2020). Plant richness, turnover, and evolutionary diversity track gradients of stability and ecological opportunity in a megadiversity center. Proceedings of the National Academies of Sciences of the United States of America, 117, 20027–20037. 10.1073/pnas.1915646117 PMC7443951 32759210

[eva13185-bib-0018] Cooperative Governance and Traditional Affairs (2016). Integrated urban development framework: A new deal for South African cities and towns.

[eva13185-bib-0019] Cowling, R. M. , Bradshaw, P. L. , Colville, J. F. , & Forest, F. (2017). Levyns’ Law: Explaining the evolution of a remarkable longitudinal gradient in Cape plant diversity. Transactions of the Royal Society of South Africa, 72, 184–201.

[eva13185-bib-0020] Cowling, R. M. , Cartwright, C. R. , Parkington, J. E. , & Allsopp, J. (1999). Fossil wood charcoal assemblages from Elands Bay Cave, South Africa. Journal of Biogeography, 26, 367–378.

[eva13185-bib-0021] Cowling, R. M. , Holmes, P. M. , & Rebelo, A. G. (1992). Plant diversity and endemism. In R. M. Cowling (Ed.), Ecology of fynbos: Nutrients, fire and diversity (pp. 62–112). Oxford University Press.

[eva13185-bib-0022] Cowling, R. M. , & Lombard, A. T. (2002). Heterogeneity, speciation/extinction history and climate: Explaining regional plant diversity patterns in the Cape Floristic Region. Diversity and Distributions, 8, 163–179.

[eva13185-bib-0023] Cowling, R. M. , Ojeda, F. , Lamont, B. B. , Rundel, P. W. , & Lechmere‐Oertel, R. (2005). Rainfall reliability, a neglected factor in explaining convergence and divergence of plant traits in fire‐prone ecosystems. Global Ecology and Biogeography, 14, 509–519.

[eva13185-bib-0024] Cowling, R. M. , & Potts, A. J. (2015). Climatic, edaphic and fire regime determinants of biome boundaries in the eastern Cape Floristic Region. South African Journal of Botany, 101, 73–81.

[eva13185-bib-0025] Cowling, R. M. , Potts, A. J. , Bradshaw, P. L. , Colville, J. , Arianoutsou, M. , Ferrier, S. , Forest, F. , Fyllas, N. M. , Hopper, S. D. , Ojeda, F. , Proches, Ş. , Smith, R. J. , Rundel, P. W. , Vassilakis, E. , & Zutta, B. R. (2015). Variation in plant diversity in Mediterranean‐climate ecosystems: The role of climatic and topographical stability. Journal of Biogeography, 42, 552–564.

[eva13185-bib-0026] Cowling, R. M. , Proches, S. , & Partridge, T. C. (2009). Explaining the uniqueness of the CFR: Incorporating geomorphic evolution as a factor for explaining its diversification. Molecular Phylogenetics and Evolution, 51, 64–74. 18691908 10.1016/j.ympev.2008.05.034

[eva13185-bib-0027] Cowling, R. M. , Richardson, D. M. , Schulze, R. J. , Hoffman, M. T. , Midgley, J. J. , & Hilton‐Taylor, C. (1997). Species diversity at the regional scale. In R. M. Cowling , D. M. Richardson , & S. M. Pierce (Eds.), Vegetation of Southern Africa (pp. 447–473). Cambridge University Press.

[eva13185-bib-0028] Cowling, R. M. , Rundel, P. W. , Desmet, P. G. , & Esler, K. J. (1998). Extraordinarily high regional‐scale plant diversity in southern African arid lands: Subcontinental and global comparisons. Diversity and Distributions, 4, 27–36.

[eva13185-bib-0029] Cowling, R. M. , Rundel, P. W. , Lamont, B. B. , Arroyo, M. K. , & Arianoutsou, M. (1996). Plant diversity in Mediterranean climate regions. Trends in Ecology and Evolution, 11, 362–366. 21237880 10.1016/0169-5347(96)10044-6

[eva13185-bib-0030] Daniels, S. R. , Hofmeyr, M. D. , Henen, B. T. , & Crandall, K. A. (2007). Living with the genetic signature of Miocene induced change: Evidence from the phylogeographic structure of the endemic angulate tortoise *Chersina angulata* . Molecular Phylogenetics and Evolution, 45, 915–926. 17936644 10.1016/j.ympev.2007.08.010

[eva13185-bib-0031] Des Roches, S. , Brans, K. I. , Lambert, M. , Rivkin, L. R. , Savage, A. , Schell, C. J. , Correa, C. , De Meester, L. , Diamond, S. E. , Govaert, L. , Grimm, N. B. , Harris, N. C. , Hendry, A. P. , Johnson, M. T. J. , Munshi‐South, J. , Palkovacs, E. P. , Szulkin, M. , Urban, M. C. , Verrelli, B. C. , & Alberti, M. (2020). Socio‐eco‐evolutionary dynamics in cities. Evolutionary Applications. 10.1111/eva.13065 PMC7819562 33519968

[eva13185-bib-0032] Drummond, A. J. , & Rambaut, A. (2007). BEAST: Bayesian evolutionary analysis by sampling trees. BMC Evolutionary Biology, 7, 214. 17996036 10.1186/1471-2148-7-214 PMC2247476

[eva13185-bib-0033] Dyer, R. J. (2007). The evolution of genetic topologies. Theoretical Population Biology, 71, 71–79. 16919694 10.1016/j.tpb.2006.07.001

[eva13185-bib-0034] Dyer, R. J. (2015). Population graphs and landscape genetics. Annual Review of Ecology, Evolution, and Systematics, 46, 327–342.

[eva13185-bib-0035] Dyer, R. J. (2016). gstudio: Tools related to the spatial analysis of genetic marker data. R package version 1.

[eva13185-bib-0036] Dyer, R. J. (2017). popgraph: This is an R package that constructs and manipulates population graphs. R package version 1.

[eva13185-bib-0037] Dyer, R. J. , Chan, D. M. , Gardiakos, V. A. , & Meadows, C. A. (2012). Pollination Graphs: Quantifying pollen pool covariance networks and the influence of intervening landscape on genetic connectivity in the North American understory tree, *Cornus florida* . Landscape Ecology, 27, 239–251.

[eva13185-bib-0038] Dyer, R. J. , Nason, J. D. , & Garrick, R. C. (2010). Landscape modelling of gene flow: Improved power using conditional genetic distance from the topology of population networks. Molecular Ecology, 19, 3746–3759. 20723052 10.1111/j.1365-294X.2010.04748.x

[eva13185-bib-0039] Eimanifar, A. , Kimball, R. T. , Braun, E. L. , & Ellis, J. D. (2018). Mitochondrial genome diversity and population structure of two western honey bee subspecies in the Republic of South Africa. Scientific Reports, 8, 1333. 29358597 10.1038/s41598-018-19759-3 PMC5778041

[eva13185-bib-0040] Feliner, G. N. (2014). Patterns and processes in plant phylogeography in the Mediterranean Basin. A review. Perspectives in Plant Ecology, Evolution and Systematics, 16, 265–278.

[eva13185-bib-0041] Flombaum, P. , & Sala, O. E. (2008). Higher effect of plant species diversity on productivity in natural than artificial ecosystems. Proceedings of the National Academy of Sciences of the United States of America, 105, 6087–6090. 18427124 10.1073/pnas.0704801105 PMC2329694

[eva13185-bib-0042] Forest, F. , Colville, J. F. , & Cowling, R. M. (2018). Evolutionary diversity patterns in the Cape flora of South Africa. In: R. A. Scherson , & D. P. Faith (Eds), Phylogenetic diversity (pp. 167–187). Springer Nature.

[eva13185-bib-0043] Frankham, R. (2005). Genetics and extinction. Biological Conservation, 126(2), 131–140.

[eva13185-bib-0044] Fusco, N. A. , Pehek, E. , & Munshi‐South, J. (2020). Urbanization reduces gene flow but not genetic diversity of stream salamander populations in the New York City metropolitan area. Evolutionary Applications. 10.1111/eva.13025 PMC7819553 33519959

[eva13185-bib-0045] Gaertner, M. , Larson, B. M. H. , Irlich, U. M. , Holmes, P. M. , Stafford, L. , van Wilgen, B. W. , & Richardson, D. M. (2016). Managing invasive species in cities: A framework from Cape Town, South Africa. Landscape and Urban Planning, 151, 1–9.

[eva13185-bib-0046] GEOTERRAIMAGE (2017). 2013–2014 South African national land data user report and metadata. Retrieved from https://egis.environment.gov.za/national_land_cover_data_sa.

[eva13185-bib-0047] Goldblatt, P. (1978). An analysis of the flora of southern Africa: Its characteristics, relationships, and origins. Annals of the Missouri Botanical Garden, 65, 369–436.

[eva13185-bib-0048] Goldblatt, P. (1997). Floristic diversity in the Cape Flora of South Africa. Biodiversity and Conservation, 6, 359–377.

[eva13185-bib-0049] Gonthier, D. J. , Ennis, K. K. , Farinas, S. , Hsieh, H.‐Y. , Iverson, A. L. , Batáry, P. , Rudolphi, J. , Tscharntke, T. , Cardinale, B. J. , & Perfecto, I. (2014). Biodiversity conservation in agriculture requires a multi‐scale approach. Proceedings of the Royal Society B: Biological Sciences, 281, 20141358. 10.1098/rspb.2014.1358 PMC4132690 25100703

[eva13185-bib-0050] Gonzalez‐Serna, M. J. , Cordero, P. J. , & Ortego, J. (2019). Spatiotemporally explicit demographic modelling supports a joint effect of historical barriers to dispersal and contemporary landscape composition on structuring genomic variation in a red‐listed grasshopper. Molecular Ecology, 28, 2155–2172. 30937976 10.1111/mec.15086

[eva13185-bib-0051] Graham, M. (2017). Postcolonial nature conservation in practice: the everyday challenges of on‐ground urban nature conservation, Cape Town, South Africa. GeoJournal, 82, 43–62.

[eva13185-bib-0052] Hardy, C. R. (2006). Reconstructing ancestral ecologies: Challenges and solutions. Diversity and Distributions, 12, 7–19.

[eva13185-bib-0053] Hattingh, V. , & Giliomee, J. H. (1989). Pollination of certain Leucadendron species (Proteaceae). South African Journal of Botany, 55, 387–393.

[eva13185-bib-0054] Hijmans, R. J. , & van Etten, J. (2012). Raster: Geographic analysis and modeling with raster data. R package version 2.0‐12.

[eva13185-bib-0055] Hoffmann, V. , Verboom, G. A. , & Cotterill, F. P. D. (2015). Dated plant phylogenies resolve Neogene climate and landscape evolution in the Cape Floristic Region. PLoS One, 10, e0137847. 26422465 10.1371/journal.pone.0137847 PMC4589284

[eva13185-bib-0056] Holderegger, R. , & Di Giulio, M. (2010). The genetic effects of roads: A review of empirical evidence. Basic and Applied Ecology, 11(6), 522–531.

[eva13185-bib-0057] Horn, A. , & Van Eeden, A. (2018). Measuring sprawl in the Western Cape Province, South Africa: An urban sprawl index for comparative purposes. Regional Science Policy and Practice, 10, 15–23.

[eva13185-bib-0058] Hudson, R. R. (2000). A new statistic for detecting genetic differentiation. Genetics, 155, 2011–2014. 10924493 10.1093/genetics/155.4.2011 PMC1461195

[eva13185-bib-0059] Hudson, R. R. , Slatkin, M. , & Maddison, W. P. (1992). Estimating levels of gene flow from DNA sequence data. Gentics, 13(2), 583–589. 10.1093/genetics/132.2.583 PMC1205159 1427045

[eva13185-bib-0060] Huntley, B. , Collingham, Y. C. , Singarayer, J. S. , Valdes, P. J. , Barnard, P. , Midgley, G. F. , Altwegg, R. , & Ohlemuller, R. (2016). Explaining patterns of avian diversity and endemicity: Climate and biomes of southern Africa over the last 140,000 years. Journal of Biogeography, 43, 874–886.

[eva13185-bib-0061] Johnson, M. T. J. , & Munshi‐South, J. (2017). Evolution of life in urban environments. Science, 358, eaam8327. 29097520 10.1126/science.aam8327

[eva13185-bib-0062] Jung, M. , Rowhani, P. , & Scharlemann, J. P. W. (2019). Impacts of past abrupt land change on local biodiversity globally. Nature Communications, 10, 5474. 10.1038/s41467-019-13452-3 PMC6888856 31792206

[eva13185-bib-0063] Kareiva, P. , Watts, S. , McDonald, R. , & Boucher, T. (2007). Domesticated nature: Shaping landscapes and ecosystems for human welfare. Science, 316, 1866–1869. 17600209 10.1126/science.1140170

[eva13185-bib-0064] Kearse, M. , Moir, R. , Wilson, A. , Stones‐Havas, S. , Cheung, M. , Sturrock, S. , Buxton, S. , Cooper, A. , Markowitz, S. , Duran, C. , Thierer, T. , Ashton, B. , Meintjes, P. , & Drummond, A. (2012). Geneious Basic: An integrated and extendable desktop software platform for the organization and analysis of sequence data. Bioinformatics, 28, 1647–1649. 22543367 10.1093/bioinformatics/bts199 PMC3371832

[eva13185-bib-0065] Keyghobadi, N. (2007). The genetic implications of habitat fragmentation for animals. Canadian Journal of Zoology‐Revue, 85, 1049.

[eva13185-bib-0066] Kopelman, N. M. , Mayzel, J. , Jakobsson, M. , Rosenberg, N. A. , & Mayrose, I. (2015). Clumpak: A program for identifying clustering modes and packaging population structure inferences across K. Molecular Ecology Resources, 15, 1179–1191. 25684545 10.1111/1755-0998.12387 PMC4534335

[eva13185-bib-0067] Kumar, S. , Stecher, G. , & Tamura, K. (2016). MEGA7: Molecular Evolutionary Genetics Analysis Version 7.0 for Bigger Datasets. Molecular Biology and Evolution, 33, 1870–1874. 27004904 10.1093/molbev/msw054 PMC8210823

[eva13185-bib-0068] Latimer, A. M. , Silander, J. A. , & Cowling, R. M. (2005). Neutral ecological theory reveals isolation and rapid speciation in a biodiversity hot spot. Science, 309, 1722–1725. 16151011 10.1126/science.1115576

[eva13185-bib-0069] Latimer, A. M. , Wu, S. S. , Gelfand, A. E. , & Silander, J. A. (2006). Building statistical models to analyze species distributions. Ecological Applications, 16, 33–50. 16705959 10.1890/04-0609

[eva13185-bib-0070] Leigh, D. M. , Hendry, A. P. , Vázquez‐Domínguez, E. , & Friesen, V. L. (2019). Estimated six per cent loss of genetic variation in wild populations since the industrial revolution. Evolutionary Applications, 12, 1505–1512. 31462910 10.1111/eva.12810 PMC6708419

[eva13185-bib-0071] Lexer, C. , Mangili, S. , Bossolini, E. , Forest, F. , Stölting, K. N. , Pearman, P. B. , Zimmermann, N. E. , & Salamin, N. (2013). ‘Next generation’ biogeography: Towards understanding the drivers of species diversification and persistence. Journal of Biogeography, 40, 1013–1022.

[eva13185-bib-0072] Lexer, C. , Wüest, R. O. , Mangili, S. , Heuertz, M. , Stölting, K. N. , Pearman, P. B. , Forest, F. , Salamin, N. , Zimmermann, N. E. , & Bossolini, E. (2014). Genomics of the divergence continuum in an African plant biodiversity hotspot, I: Drivers of population divergence in Restio capensis (Restionaceae). Molecular Ecology, 23, 4373–4386. 25065899 10.1111/mec.12870

[eva13185-bib-0073] Linder, H. P. (2003). The radiation of the Cape Flora, Southern Africa. Biological Reviews, 78, 597–638. 14700393 10.1017/s1464793103006171

[eva13185-bib-0074] Linder, H. P. (2005). Evolution of diversity: The Cape flora. Trends in Plant Science, 10, 536–541. 16213780 10.1016/j.tplants.2005.09.006

[eva13185-bib-0075] Linder, H. P. (2006). Investigating the evolution of floras: Problems and progress ‐ an introduction. Diversity and Distributions, 12, 3–5.

[eva13185-bib-0076] Linder, H. P. (2008). Plant species radiations: Where, when, why? Philosophical Transactions of the Royal Society B: Biological Sciences, 363, 3097–3105. 10.1098/rstb.2008.0075 PMC2607312 18579472

[eva13185-bib-0077] Linder, H. P. , & Hardy, C. R. (2004). Evolution of the species‐rich Cape Flora. Philosophical Transactions of the Royal Society B: Biological Sciences, 359, 1623–1632. 10.1098/rstb.2004.1534 PMC1693430 15519977

[eva13185-bib-0078] Lopez, S. , Rousset, F. , Shaw, F. H. , Shaw, R. G. , & Ronce, O. (2008). Migration load in plants: Role of pollen and seed dispersal in heterogeneous landscapes. Journal of Evolutionary Biology, 21, 294–309. 17995948 10.1111/j.1420-9101.2007.01442.x

[eva13185-bib-0079] Manne, L. L. , Williams, P. H. , Midgley, G. F. , Thuiller, W. , Rebelo, A. G. , & Hannah, L. (2007). Spatial and temporal variation in species‐area relationships in the fynbos biological hotspot. Ecography, 30, 852–861.

[eva13185-bib-0080] Marean, C. W. (2016). The transition to foraging for dense and predictable resources and its impact on the evolution of modern humans. Philosophical Transactions of the Royal Society B: Biological Sciences, 371, 20150239. 10.1098/rstb.2015.0239 PMC4920296 27298470

[eva13185-bib-0081] Marean, C. W. , Cawthra, H. C. , Cowling, R. M. , Esler, K. J. , Fisher, E. , Milewski, A. V. , Potts, A. G. , Singels, E. , & de Vynck, J. (2014). Stone Age people in a changing South African Greater Cape Floristic Region. In: N. Allsopp , J. C. Colville , & G. A. Verboom (Eds), Fynbos: Ecology, evolution, and conservation of a megadiverse region (pp. 164–199). Oxford University Press.

[eva13185-bib-0082] McDonald, J. H. , Verrelli, B. C. , & Geyer, L. B. (1996). Lack of geographic variation in anonymous nuclear polymorphisms in the American oyster, *Crassostrea virginica* . Molecular Biology and Evolution, 13, 1114–1118. 8865664 10.1093/oxfordjournals.molbev.a025673

[eva13185-bib-0083] McKinney, M. (2006). Urbanization as a major cause of biotic homogenization. Biological Conservation, 127, 247–260.

[eva13185-bib-0084] McRae, B. H. (2006). Isolation by resistance. Evolution, 60(8), 1551–1561. 17017056

[eva13185-bib-0085] Miles, L. S. , Dyer, R. J. , & Verrelli, B. C. (2018). Urban hubs of connectivity: Contrasting patterns of gene flow within and among cities in the western black widow spider. Proceedings of the Royal Society B, 285, 20181224. 30068686 10.1098/rspb.2018.1224 PMC6111156

[eva13185-bib-0086] Miles, L. S. , Johnson, J. C. , Dyer, R. J. , & Verrelli, B. C. (2018). Urbanization as a facilitator of gene flow in a human health pest. Molecular Ecology, 27, 3219–3230. 10.1111/mec.14783 29972610

[eva13185-bib-0087] Miles, L. S. , Rivkin, L. R. , Johnson, M. T. J. , Munshi‐South, J. , & Verrelli, B. C. (2019). Gene flow and genetic drift in urban environments. Molecular Ecology, 28, 4138–4151. 31482608 10.1111/mec.15221

[eva13185-bib-0088] Moreno‐Mateos, D. , Barbier, E. , Jones, P. , Jones, H. P. , Aronson, J. , López‐López, J. A. , McCrackin, M. L. , Meli, P. , Montoya, D. , & Rey Benayas, J. M. (2017). Anthropogenic ecosystem disturbance and the recovery debt. Nature Communications, 8, 14163. 10.1038/ncomms14163 PMC5263871 28106039

[eva13185-bib-0089] Morueta‐Holme, N. , Enquist, B. J. , McGill, B. J. , Boyle, B. , Jorgensen, P. M. , Ott, J. E. , Peet, R. K. , Šimova, I. , Sloat, L. L. , Thiers, B. , & Violle, C. (2013). Habitat area and climate stability determine geographical variation in plant species range sizes. Ecology Letters, 16, 1446–1454. 24119177 10.1111/ele.12184 PMC4068282

[eva13185-bib-0090] Myers, N. , Mittermeier, R. A. , Mittermeier, C. G. , da Fonseca, G. A. B. , & Kent, J. (2000). Biodiversity hotspots for conservation priorities. Nature, 403, 853–858. 10706275 10.1038/35002501

[eva13185-bib-0091] Newbold, T. , Hudson, L. N. , Hill, S. L. L. , Contu, S. , Lysenko, I. , Senior, R. A. , Börger, L. , Bennett, D. J. , Choimes, A. , Collen, B. , Day, J. , De Palma, A. , Díaz, S. , Echeverria‐Londoño, S. , Edgar, M. J. , Feldman, A. , Garon, M. , Harrison, M. L. K. , Alhusseini, T. , … Purvis, A. (2015). Global effects of land use on local terrestrial biodiversity. Nature, 520, 45–50. 25832402 10.1038/nature14324

[eva13185-bib-0092] Olivieri, I. , Tonnabel, J. , Ronce, O. , & Mignot, A. (2015). Why evolution matters for species conservation: Perspectives from three case studies of plant metapopulations. Evolutionary Applications, 9, 196–211. 27087848 10.1111/eva.12336 PMC4780382

[eva13185-bib-0093] Pfeifer, B. , Wittelsbuerger, U. , Ramos‐Onsins, S. E. , & Lercher, M. J. (2014). PopGenome: An efficient swiss army knife for population genomic analyses in R. Molecular Biology and Evolution, 31, 1929–1936. 24739305 10.1093/molbev/msu136 PMC4069620

[eva13185-bib-0094] Pharmawati, M. , Yan, G. , Sedgley, R. , & Finnegan, P. M. (2004). Chloroplast DNA inheritance and variation in Leucadendron species (Proteaceae) as revealed by PCR‐RFLP. Theoretical Applied Genetics, 109, 1694–1701. 15365629 10.1007/s00122-004-1800-z

[eva13185-bib-0095] Pinheiro, J. , Bates, D. , DebRoy, S. S. , & Sarkar, D. (2013). Nlme: linear and nonlinear mixed effects models. R package version 31‐110.

[eva13185-bib-0096] Pirie, M. D. , Oliver, E. G. , Mugrabi de Kuppler, A. , Gehrke, B. , Le Maitre, N. C. , Kandziora, M. , & Bellstedt, D. U. (2016). The biodiversity hotspot as evolutionary hot‐bed: Spectacular radiation of Erica in the Cape Floristic Region. BMC Evolutionary Biology, 16, 190. 27639849 10.1186/s12862-016-0764-3 PMC5027107

[eva13185-bib-0097] Portik, D. M. , Bauer, A. M. , & Jackman, T. R. (2011). Bridging the gap: Western rock skinks (*Trachylepis sulcata*) have a short history in South Africa. Molecular Ecology, 20, 1744–1758. 21371148 10.1111/j.1365-294X.2011.05047.x

[eva13185-bib-0098] Potts, A. J. (2017). Catchments catch all in South African coastal lowlands: topography and palaeoclimate restricted gene flow in *Nymania capensis* (Meliaceae)—a multilocus phylogeographic and distribution modelling approach. PeerJ, 5, e2965. 28168122 10.7717/peerj.2965 PMC5289106

[eva13185-bib-0099] Potts, A. J. , Hedderson, T. A. , Franklin, J. , & Cowling, R. M. (2013). The Last Glacial Maximum distribution of South African subtropical thicket inferred from community distribution modelling. Journal of Biogeography, 40, 310–322.

[eva13185-bib-0100] Potts, A. J. , Moncrieff, G. R. , Bond, W. J. , & Cowling, R. M. (2015). An operational framework for biome boundary research with examples from South Africa. South African Journal of Botany, 101, 5–15.

[eva13185-bib-0101] Proches, S. , Cowling, R. M. , Goldblatt, P. , Manning, J. C. , & Snijman, D. A. (2006). An overview of the Cape geophytes. Biological Journal of the Linnean Society, 87, 27–43.

[eva13185-bib-0102] Prunier, R. , & Holsinger, K. E. (2010). Was it an explosion? Using population genetics to explore the dynamics of a recent radiation within Protea. Molecular Ecology, 19, 3968–3980. 20723047 10.1111/j.1365-294X.2010.04779.x

[eva13185-bib-0103] Raj, A. , Stephens, M. , & Pritchard, J. K. (2014). fastSTRUCTURE: Variational inference of population structure in large SNP data sets. Genetics, 197, 573–589. 24700103 10.1534/genetics.114.164350 PMC4063916

[eva13185-bib-0104] Rebelo, A. G. , Holmes, P. M. , Dorse, C. , & Wood, J. (2011). Impacts of urbanization in a biodiversity hotspot: Conservation challenges in Metropolitan Cape Town. South African Journal of Botany, 77, 20–35.

[eva13185-bib-0105] Rebelo, A. G. , Mtshali, H. , & von Staden, L. (2019). Leucadendron salignum P.J.Bergius. National Assessment: Red List of South African Plants version 2020.1.

[eva13185-bib-0106] Rector, A. L. , & Verrelli, B. C. (2010). Glacial cycling, large mammal community composition, and trophic adaptations in the Western Cape, South Africa. Journal of Human Evolution, 58, 90–102. 19914679 10.1016/j.jhevol.2009.09.004

[eva13185-bib-0107] Richardson, J. E. , Weitz, F. M. , Fay, M. F. , Cronk, Q. C. B. , Linder, H. P. , Reeves, G. , & Chase, M. W. (2001). Rapid and recent origin of species richness in the Cape Flora of South Africa. Nature, 412, 181–183. 11449273 10.1038/35084067

[eva13185-bib-0108] Rivkin, L. R. , Santangelo, J. S. , Alberti, M. , Aronson, M. F. J. , de Keyzer, C. W. , Diamond, S. E. , Fortin, M. J. , Frazee, L. J. , Gorton, A. J. , Hendry, A. P. , Liu, Y. , Losos, J. B. , MacIvor, J. S. , Martin, R. A. , McDonnell, M. J. , Miles, L. S. , Munshi‐South, J. , Ness, R. W. , Newman, A. E. M. , … Johnson, M. T. J. (2018). A roadmap for urban evolutionary ecology. Evolutionary Applications, 12, 384–398. 30828362 10.1111/eva.12734 PMC6383741

[eva13185-bib-0109] Rosenberg, M. S. , & Anderson, C. D. (2011). PASSaGE. Pattern Analysis, Spatial Statistics and Geographic Exegesis. Version 2. Methods in Ecology and Evolution, 2, 229–232.

[eva13185-bib-0110] Rosenberg, N. A. (2004). Distruct: A program for the graphical display of population structure. Molecular Ecology Notes, 4, 137–138.

[eva13185-bib-0111] Rost, S. , Pelz, H. J. , Menzel, S. , MacNicoll, A. D. , Leon, V. , Song, K. J. , Jakel, T. , Oldenburg, J. , & Muller, C. R. (2009). Novel mutations in the VKORC1 gene of wild rats and mice: a response to 50 years of selection pressure by warfarin? BMC Genetics, 10, 9. 19200363 10.1186/1471-2156-10-4 PMC2644709

[eva13185-bib-0112] Rouget, M. , Richardson, D. M. , Cowling, R. M. , Lloyd, J. W. , & Lombard, A. T. (2003). Current patterns of habitat transformation and future threats to biodiversity in terrestrial ecosystems of the Cape Floristic Region, South Africa. Biological Conservation, 112, 63–85.

[eva13185-bib-0113] Row, J. R. , Knick, S. T. , Oyler‐McCance, S. J. , Lougheed, S. C. , & Fedy, B. C. (2017). Developing approaches for linear mixed modeling in landscape genetics through landscape‐directed dispersal simulations. Ecology and Evolution, 7, 3751–3761. 28616172 10.1002/ece3.2825 PMC5468135

[eva13185-bib-0114] Rundel, P. W. , Arroyo, M. T. K. , Cowling, R. M. , Keeley, J. E. , Lamont, B. B. , & Vargas, P. (2016). Mediterranean biomes: Evolution of their vegetation, floras, and climate. Annual Review of Ecology, Evolution, and Systematics, 47, 383–407.

[eva13185-bib-0115] Rymer, P. D. , Manning, J. C. , Goldblatt, P. , Powell, M. P. , & Savolainen, V. (2010). Evidence of recent and continuous speciation in a biodiversity hotspot: A population genetic approach in southern African gladioli (Gladiolus; Iridaceae). Molecular Ecology, 19, 4765–4782. 20735739 10.1111/j.1365-294X.2010.04794.x

[eva13185-bib-0116] Sala, O. E. , Chapin, F. S. 3rd , Armesto, J. J. , Berlow, E. , Bloomfield, J. , Dirzo, R. , Huber‐Sanwald, E. , Huenneke, L. F. , Jackson, R. B. , Kinzig, A. , Leemans, R. , Lodge, D. M. , Mooney, H. A. , Oesterheld, M. , Poff, N. L. , Sykes, M. T. , Walker, B. H. , Walker, M. , & Wall, D. H. (2000). Global biodiversity scenarios for the year 2100. Science, 287, 1770–1774. 10710299 10.1126/science.287.5459.1770

[eva13185-bib-0117] Sauquet, H. , Weston, P. H. , Barker, N. P. , Anderson, C. L. , Cantrill, D. J. , & Savolainen, V. (2009). Using fossils and molecular data to reveal the origins of the Cape Proteas (Subfamily Proteoideae). Molecular Phylogenetics and Evolution, 51, 31–43. 19135535 10.1016/j.ympev.2008.12.013

[eva13185-bib-0118] Schnitzler, J. , Barraclough, T. J. , Boatwright, J. S. , Goldblatt, P. , Manning, J. C. , Powell, M. P. , Rebelo, A. G. , & Savolainen, V. (2011). Causes of plant diversification in the Cape biodiversity hotspot of South Africa. Systematic Biology, 60, 343–357. 21362644 10.1093/sysbio/syr006

[eva13185-bib-0119] Schulze, R. E. (1997) South African Atlas of Agrohydrology and Climatology. TT82/96, Water Research Commission.

[eva13185-bib-0120] Slatkin, M. (1993). Isolation by distance in equilibrium and nonequilibrium populations. Evolution, 47, 264–279. 28568097 10.1111/j.1558-5646.1993.tb01215.x

[eva13185-bib-0121] Slingsby, J. A. , Moncrieff, G. R. , & Wilson, A. M. (2020). Near‐real time forecasting and change detection for an open ecosystem with complex natural dynamics. ISPRS Journal of Photogrammetry and Remote Sensing, 166, 15–25.

[eva13185-bib-0122] Slingsby, J. , & Verboom, G. A. (2006). Phylogenetic relatedness limits co‐occurrence at fine spatial scales: Evidence from the Schoenoid Sedges (Cyperaceae: Schoeneae) of the Cape Floristic Region, South Africa. The American Naturalist, 168, 14–27. 10.1086/505158 16874612

[eva13185-bib-0123] Smit, H. A. , Robinson, T. J. , & Van Vuuren, B. J. (2007). Coalescence methods reveal the impact of vicariance on the spatial genetic structure of *Elephantulus edwardii* (Afrotheria, Macroscelidea). Molecular Ecology, 16, 2680–2692. 17594439 10.1111/j.1365-294X.2007.03334.x

[eva13185-bib-0124] South African National Biodiversity Institute (2018). The Vegetation Map of South Africa, Lesotho and Swaziland. L. Mucina , M. C. Rutherford , & L. W. Powrie (Ed). Retrieved from http://bgis.sanbi.org/Projects/Detail/186, Version 2018.

[eva13185-bib-0125] Tajima, F. (1989). Statistical‐method for testing the neutral mutation hypothesis by DNA polymorphism. Genetics, 123, 585–595. 2513255 10.1093/genetics/123.3.585 PMC1203831

[eva13185-bib-0126] Thatte, P. , Chandramouli, A. , Tyagi, A. , Patel, K. , Baro, P. , Chhattani, H. , & Ramakrishnan, U. (2020). Human footprint differentially impacts genetic connectivity of four wide‐ranging mammals in a fragmented landscape. Diversity and Distributions, 26, 299–314.

[eva13185-bib-0127] Thuiller, W. , Midgley, G. F. , Rouget, M. , & Cowling, R. M. (2006). Predicting patterns of plant species richness in megadiverse South Africa. Ecography, 29, 733–744.

[eva13185-bib-0128] Tolley, K. A. , Burger, M. , Turner, A. A. , & Matthee, C. A. (2006). Biogeographical patterns and phylogeography of dwarf chameleons (Bradypodion) in an African biodiversity hotspot. Molecular Ecology, 15, 781–793. 16499702 10.1111/j.1365-294X.2006.02836.x

[eva13185-bib-0129] Tonnabel, J. , Mignot, A. , Douzery, E. J. , Rebelo, A. G. , Schurr, F. M. , Midgley, J. , Illing, N. , Justy, F. , Orcel, D. , & Olivieri, I. (2014). Convergent and correlated evolution of major life‐history traits in the angiosperm genus Leucadendron (Proteaceae). Evolution, 68, 2775–2792. 24957971 10.1111/evo.12480

[eva13185-bib-0130] Underwood, E. C. , Klausmeyer, K. R. , Cox, R. L. , Busby, S. M. , Morrison, S. A. , & Shaw, M. R. (2009). Expanding the global network of protected areas to save the imperiled mediterranean biome. Conservation Biology, 23, 43–52. 18950475 10.1111/j.1523-1739.2008.01072.x

[eva13185-bib-0131] Valente, L. M. , Reeves, G. , Schnitzler, J. , Mason, I. P. , Fay, M. F. , Rebelo, A. G. , & Barraclough, T. G. (2010). Diversification of the African genus Protea (Proteaceae) in the Cape biodiversity hotspot and beyond: Equal rates in different biomes. Evolution, 64, 745–759. 19804404 10.1111/j.1558-5646.2009.00856.x

[eva13185-bib-0132] Van Hooft, W. F. , Groen, A. F. , & Prins, H. T. (2002). Phylogeography of the African buffalo based on mitochondrial and Y‐chromosomal loci: Pleistocene origin and population expansion of the Cape buffalo subspecies. Molecular Ecology, 11, 267–279. 11856427 10.1046/j.1365-294x.2002.01429.x

[eva13185-bib-0133] Van Wyk, B.‐E. (2011). The potential of South African plants in the development of new medicinal products. South African Journal of Botany, 77, 812–829.

[eva13185-bib-0134] Verboom, G. A. , Archibald, J. K. , Bakker, F. T. , Bellstedt, D. U. , Conrad, F. , Dreyer, L. L. , Forest, F. , Galley, C. , Goldblatt, P. , Henning, J. F. , Mummenhoff, K. , Linder, H. P. , Muasya, A. M. , Oberlander, K. C. , Savolainen, V. , Snijman, D. A. , van der Niet, T. , & Nowell, T. L. (2009). Origin and diversification of the Greater Cape flora: Ancient species repository, hot‐bed of recent radiation, or both? Molecular Phylogenetics and Evolution, 51, 44–53. 18411064 10.1016/j.ympev.2008.01.037

[eva13185-bib-0135] Verboom, G. A. , Bergh, N. G. , Haiden, S. A. , Hoffmann, V. , & Britton, M. N. (2015). Topography as a driver of diversification in the Cape Floristic Region of South Africa. New Phytologist, 207, 368–376. 10.1111/nph.13342 25708902

[eva13185-bib-0136] Vlok, J. H. J. , Euston‐Brown, D. I. W. , & Cowling, R. M. (2003). Acocks’ Valley Bushveld 50 years on: New perspectives on the delimitation, characterisation and origin of thicket vegetation. South African Journal of Botany, 69, 27–51.

[eva13185-bib-0137] Wasserman, S. , & Faust, K. (1994). Social network analysis: Methods and applications. Cambridge University Press.

[eva13185-bib-0138] Welsford, M. R. , Hobbhahn, N. , Midgley, J. J. , & Johnson, S. D. (2016). Floral trait evolution associated with shifts between insect and wind pollination in the dioecious genus Leucadendron (Proteaceae). Evolution, 70, 126–139. 26593965 10.1111/evo.12821

[eva13185-bib-0139] Welsford, M. R. , Midgley, J. J. , & Johnson, S. D. (2014). Experimental evaluation of insect pollination versus wind pollination in Leucadendron (Proteaceae). International Journal of Plant Science, 175, 296–306.

[eva13185-bib-0140] Winchell, K. M. , Reynolds, R. G. , Prado‐Irwin, S. R. , Puente‐Rolon, A. R. , & Revell, L. J. (2016). Phenotypic shifts in urban areas in the tropical lizard *Anolis cristatellus* . Evolution, 70, 1009–1022. 27074746 10.1111/evo.12925

[eva13185-bib-0141] Wolfe, K. H. , Li, W. H. , & Sharp, P. M. (1987). Rates of nucleotide substitution vary greatly among plant mitochondrial, chloroplast, and nuclear DNAs. Proceedings of the National Academy of Sciences of the United States of America, 84, 9054–9058. 3480529 10.1073/pnas.84.24.9054 PMC299690

[eva13185-bib-0142] Wright, S. W. (1982). The shifting balance theory and macroevolution. Annual Review of Genetics, 16, 1. 10.1146/annurev.ge.16.120182.000245 6760797

