# Supplementary material for: Evolutionary stability, landscape heterogeneity, and human land‐usage shape population genetic connectivity in the Cape Floristic Region biodiversity hotspot
Source: Evol Appl. 2021 Jan 13;14(4):1109–23. doi: 10.1111/eva.13185 (PMC8061270; doi:10.1111/eva.13185)
Supplement: Supplementary file 1 — Supplementary Material [file EVA-14-1109-s001.docx]

**SUPPORTING INFORMATION**

**Supporting Information S1: Data Collection**

For the chloroplast (cp) DNA dataset, we designed dozens of degenerate PCR primers that targeted many chloroplast regions typically used for plant phylogenetic analyses (Hu et al., 2000; Birky, 2001), but that spanned non-coding regions where putatively neutral variation is more likely to occur. For the nuclear (nu) DNA dataset, we used ISSR PCR primers selected from Bergh et al. (2007), but in many multiplex PCR combinations to randomly generate hundreds of independent nucleotide sequence fragments, which again, has the intent to target putatively neutral variation. This approach did not intend to target ISSR fragments, but instead used these previously designed primers to generate full nucleotide sequence fragments. We used a randomly sampled subset of individuals, where PCR conditions varied temperature across degenerate primer combinations, and fragments of ~250-500 bp were size-selected from 2% agarose electrophoresis gels from each individual for each of the chloroplast and nuclear marker sets. Excised fragments were each eluted from gel via suspension in water overnight.

Hundreds of PCR fragments (generated from degenerate primer combinations as above), from each of the chloroplast and nuclear datasets were barcoded using the Nextera XT DNA sample preparation kit (Illumina, Inc.). Barcoded fragments were then pooled and DNA sequences were collected on an Illumina MiSeq (250bp, paired end). The resulting fasta files were aligned and visualized using the default parameters of the MUSCLE alignment in the program Geneious v. 4.8.4 (Kearse et al., 2012). For the cpDNA dataset, we filtered out any potential multiple copy or nuclear fragments (e.g., evidence of heterozygous sites), whereas, for the nuDNA dataset, we filtered out any potential multiple copy or haploid fragments (e.g., excess of SNP site homozygosity that would violate Hardy-Weinberg expectations).

Based on this first round of sequencing using degenerate and random primer generation, new PCR primers were generated to amplify nuclear and chloroplast PCR fragments from our 306 individuals in multiplex reactions. From this second round of PCR, the cpDNA and nuDNA products were directly barcoded using the Nextera XT DNA sample preparation kit (Illumina, Inc.). Samples were pooled (by groups of 96 unique barcodes) and DNA sequences were collected on an Illumina MiSeq (250bp, paired end). Variant and SNP calling was performed using the CLC Genomics Workbench v. 5.5 (CLC Bio, Cambridge, MA) using a minimum PHRED quality threshold of 30, a minimum variant coverage of 10 reads, and a variant probability of 90. All data were again organized, aligned, and visualized in Geneious (Kearse et al. 2012).

Multiple levels of post-sequence curation was performed to provide a conservative dataset that best reflected neutrality and demographic processes. One consideration is that sequence variation within the chloroplast genome is expected to be inherently linked. However, this inherent linkage should not be the case in the nuclear genome, and thus, where unusual blocks of LD were identified in the nuclear dataset (estimated correlations as r^2^ among SNPs within fragments), we removed these fragments from the analysis (fewer than 5%). As above, for the cpDNA dataset, we filtered out any potential multiple copy or nuclear fragments (e.g., evidence of heterozygous sites), whereas, for the nuDNA dataset, we filtered out any potential multiple copy or haploid fragments (e.g., excess of SNP site homozygosity that would violate Hardy-Weinberg expectations). Finally, we filtered out all fragments with highly repetitive regions (i.e., microsatellites or complex regions), and performed a blastn query (Genbank) to filter out any evidence of putative coding regions with open reading frames.

Variable sites were retained in our dataset only if they could be resolved for all individuals, (i.e., no “missing” data). This step may be considered conservative, i.e., next generation sequencing protocols often produce missing data for individuals, but which often has little impact given the total number of variants sampled across the genome is high. Thus, we were less concerned with the conservative removal of SNPs, and more concerned about the potential of spurious results and overestimates of population structure that are caused by locale-specific SNPs that could not be evaluated in all locales. Finally, all SNPs were concatenated within each of the chloroplast and nuclear datasets.

**Supporting Information S2: Phylogenetic Analyses and Dating Estimates**

We conducted several analyses to place genetic divergence among locales and lineages in an evolutionary framework. While we are interested in the phylogenetic structure among locales and lineages, we are also interested in whether we could generate date estimates for this structure. However, although prior phylogenetic tree analyses of the *Leucadendron* genus have been published (Barker et al., 2004; Tonnabel et al., 2014; Hoffmann et al., 2015), molecular clock divergence estimates have not been previously estimated for this group. Thus, we followed the approach of Valente et al. (2010), which estimated divergence times of the closely-related South African *Protea* genus using a relaxed Bayesian MCMC approach implemented in BEAST (Drummond & Rambaut, 2007). We also generated nucleotide sequence data for a closely-related outgroup *Leucadendron laureolum* (Barker et al., 2004) sampled from the Cape Peninsula (Figure S4) to align with our *L. salignum* data. With this outgroup and the approach of Valente et al. (2010), we generated a rooted *L. salignum* phylogenetic tree with date estimates for interpreting geographic and evolutionary structure.

First, we analyzed Genbank publicly-available ITS nuclear sequence data for 14 *Leucadendron* species (including *L. salignum*), and the outgroup *Serruria adscendens* (see accession information in Table S2), which are currently the only nucleotide data published for *Leucadendron* species from a previous phylogenetic analysis (Barker et al., 2004). Similar to Valente et al. (2010), our BEAST (v.1.7.5) analysis used a speciation model following a Yule process as the tree prior with the starting tree randomly generated, and branch rates were set as uncorrelated lognormal with rates estimated among branches. As a point of validation, this iteration of our calibration resulted in the same phylogenetic topology as previously published for these taxa (Barker et al., 2004; Tonnabel et al., 2014; Hoffmann et al., 2015).

Using the tree topology of the previous iteration and the stem group of *Leucadendron* estimated at 29.3-46.2 Mya based on fossil calibration by Sauquet et al. (2009), we constrained the root age to a normal distribution across this time range. Five independent runs of 5 million generations, sampling every 2000 generations, were run in BEAST for root estimates sampled within this distribution. The effective sample size (ESS) for each run was assessed using Tracer v.1.5 (Drummond & Rambaut, 2007), with the independent runs showing immediate convergence. All runs were combined using LogCombiner, with trees annotated using TreeAnnotator v.1.7.5 (Drummond & Rambaut, 2007), and visualized with FigTree (Rambaut, 2012). The divergence time between *L. salignum* and all other *Leucadendron* taxa that had the highest support from this analysis was a root estimate of 14 Mya, which was used in all dating estimates explained in the population divergence modeling below. Previous studies have shown that using alternate tree priors as well as a birth-death process in BEAST had little effect on divergence times and topologies (Valente et al., 2010). Nonetheless, our investigation of these alternate approaches and priors resulted in the same outcomes within our analysis.

         To address the need for an outgroup for our *L. salignum* population tree estimates, we sampled multiple individuals of *Leucadendron laureolum* from the Cape Peninsula (DaGama Park, located at -34.1693472, 18.4078722). To confirm its suitability as an outgroup, a preliminary analysis of cpDNA nucleotide pairwise sequence divergence between *L. laureolum* and *L.salignum* was estimated at 10-fold higher than that observed between any two *L. salignum* individuals in our dataset (θπ = 2.88% vs 0.25%, respectively). This nucleotide site divergence estimate is similar to that in Pharmawati et al. (2005). In fact, this previous study showed that cultivars of *L. salignum* form a sister group to the hybrid progeny of *L. salignum* × *L. laureolum*, which are morphologically similar and occur naturally on the sandstone flats of the Cape Peninsula (Figure S4). Thus, this previous study and our preliminary nucleotide sequence analysis implies that our sampling of *L. laureolum* is an appropriate outgroup for our *L. salignum* evolutionary tree analyses in BEAST.

         Using the molecular clock estimates from the species tree analysis above, we then performed a BEAST analysis to estimate the evolutionary topology and time divergence for each of the two *L. salignum* chloroplast and nuclear datasets. We used jMODELTEST (Posada, 2008) to estimate the appropriate model of nucleotide site evolution. From Wolfe et al. (1987), we used standard estimates of sequence divergence for cpDNA (1.0-3.0 X 10^-9^ substitutions/site/year) and nuDNA (3.0 X 10^-8^ substitutions/site/year). MCMC analyses were performed using both strict and relaxed (uncorrelated lognormal) clocks, and using the constant size, exponential growth, and Bayesian skyline population models. Rate variation was allowed by setting a normal prior distribution for the molecular clock rate (mean = 0.001, SD = 0.00001). Each BEAST profile was run 5 times for 60 million generations with the first 6 million discarded as burn-in, logging every 6000. As detailed above, runs were viewed in Tracer, and log files from each set of runs were then combined using LogCombiner and TreeAnnotator yielding a consensus tree.

**Table S1. Locale information for *Leucadendron salignum* samples**

| Locale # | Name | Abbreviation | Latitude | Longitude |
| --- | --- | --- | --- | --- |
| 1 | Vanrhyns Pass | VRP | -34.344117 | 21.867300 |
| 2 | Gifberg Pass | GIP | -31.769266 | 18.769991 |
| 3 | Pakhuis Pass | PAP | -32.144417 | 19.024917 |
| 4 | Middelberg Pass | MIP | -32.630514 | 19.152213 |
| 5 | Versfeld Pass | VEP | -32.842528 | 18.731639 |
| 6 | Gydo Pass | GYP | -33.235953 | 19.336479 |
| 7 | Grotto Bay | GBY | -33.525800 | 18.353883 |
| 8 | Worcester | WOR | -33.602550 | 19.334633 |
| 9 | Philadelphia | PHI | -33.717500 | 18.544033 |
| 10 | Cape Town | CPT | -33.916982 | 18.404876 |
| 11 | Silvermine | SVR | -34.082783 | 18.414900 |
| 12 | Stellenbosch | STL | -33.932383 | 18.876867 |
| 13 | Simons Town | SIT | -34.200633 | 18.411206 |
| 14 | Smitswinkel Bay | SWB | -34.261931 | 18.461039 |
| 15 | Pringle Bay | PRB | -34.261931 | 18.461039 |
| 16 | Franschhoek Pass | FRP | -33.914948 | 19.157012 |
| 17 | Greyton | GRY | -34.033451 | 19.607516 |
| 18 | Stanford | STA | -34.410323 | 19.588872 |
| 19 | Agulhas | AGU | -34.669667 | 19.775300 |
| 20 | Kogmanskloof Pass | KOP | -33.803773 | 20.105717 |
| 21 | Bontebok | BTK | -34.043683 | 20.469467 |
| 22 | DeHoop | DHP | -34.379367 | 20.529734 |
| 23 | Anysberg | ANB | -33.472639 | 20.582028 |
| 24 | Tradouws Pass | TRP | -33.937058 | 20.711612 |
| 25 | Heidelberg | HID | -34.014967 | 20.966033 |
| 26 | Garcia Pass | GAP | -33.941639 | 21.201873 |
| 27 | Still Bay | STB | -34.262791 | 21.371451 |
| 28 | Albertinia | ALB | -34.229251 | 21.593599 |
| 29 | Vlees Bay | VLB | -34.344117 | 21.867300 |
| 30 | Mossel Bay | MOB | -34.165919 | 22.007854 |
| 31 | Seweweekspoort | SEW | -33.386333 | 21.408050 |
| 32 | Rooiberg Pass | ROP | -33.863900 | 22.028350 |
| 33 | Cloete Pass | CLP | -33.930950 | 21.761383 |
| 34 | Swartberg Pass | SWP | -33.350967 | 22.047367 |
| 35 | Robinsons Pass | RBP | -33.863900 | 22.028350 |
| 36 | Grootbrak | GRB | -34.063192 | 22.201927 |
| 37 | Outeniqua Pass | OUP | -33.886900 | 22.399708 |
| 38 | Daskop | DAS | -33.769383 | 22.656000 |
| 39 | Blesberg | BLB | -33.407750 | 22.732306 |
| 40 | Potjiesberg Pass | POP | -33.702722 | 23.043639 |
| 41 | Plettenberg Bay | PLB | -34.013248 | 23.388419 |
| 42 | Misgund | MIS | -33.760434 | 23.482735 |
| 43 | Kougaberg | KOU | -33.675250 | 23.503300 |
| 44 | Joubertina | JOB | -33.820583 | 23.854000 |
| 45 | Nooitgedacht | NOT | -33.826917 | 24.254450 |
| 46 | Assegaaibos | AGS | -33.934554 | 24.303512 |
| 47 | East Baviaanskloof | BAV | -33.634444 | 24.469167 |
| 48 | Humansdorp | HUM | -33.959918 | 24.766698 |
| 49 | Stinkhoutberg | SHB | -33.815336 | 24.950685 |
| 50 | Suurberg Pass | SUP | -33.282627 | 25.720100 |
| 51 | Grahamstown | GRT | -33.340277 | 26.516580 |

**Table S2. Taxa and nucleotide sequence accession data for phylogenetic calibration analyses**

| Species | GenBank Accession Numbers |
| --- | --- |
| *Leucadendron nervosum* | AY692171.1 |
| *Leucadendron album* | AY692167.1 |
| *Leucadendron ericifolium* | AF508855.1 |
| *Leucadendron flexuosum* | AY692169.1 |
| *Leucadendron salignum* | AY692172.1 |
| *Leucadendron lanigerum* | AY692170.1 |
| *Leucadendron discolor* | AY692202.1 |
| *Leucadendron modestum* | AY692221.1 |
| *Leucadendron dregei* | AY692166.1 |
| *Leucadendron singulare* | AY692209.1 |
| *Leucadendron platyspermum* | AY692205.1 |
| *Leucadendron rubrum* | AY692186.1 |
| *Leucadendron argenteum* | AY692184.1 |
| *Leucadendron osbornei* | AY692168.1 |
| *Serruria adscendens (outgroup)* | AF508823.1 |

**Table S3. Nucleotide diversity estimates per locale for *Leucadendron salignum* samples**

| Locale # | Name | nuclear (%) | | chloroplast (%) | |
| --- | --- | --- | --- | --- | --- |
|  |  | Theta-S | Theta-pi | Theta-S | Theta-pi |
| 1 | Vanrhyns Pass | 0.225 | 0.583 | 0.010 | 0.020 |
| 2 | Gifberg Pass | 0.162 | 0.448 | 0.041 | 0.095 |
| 3 | Pakhuis Pass | 0.152 | 0.368 | 0.022 | 0.071 |
| 4 | Middelberg Pass | 0.278 | 0.724 | 0.038 | 0.119 |
| 5 | Versfeld Pass | 0.159 | 0.397 | 0.003 | 0.007 |
| 6 | Gydo Pass | 0.187 | 0.489 | 0.000 | 0.000 |
| 7 | Grotto Bay | 0.210 | 0.515 | 0.006 | 0.017 |
| 8 | Worcester | 0.225 | 0.545 | 0.003 | 0.007 |
| 9 | Philadelphia | 0.258 | 0.566 | 0.044 | 0.113 |
| 10 | Cape Town | 0.180 | 0.474 | 0.013 | 0.031 |
| 11 | Silvermine | 0.157 | 0.408 | 0.038 | 0.100 |
| 12 | Stellenbosch | 0.190 | 0.468 | 0.054 | 0.127 |
| 13 | Simons Town | 0.197 | 0.490 | 0.010 | 0.031 |
| 14 | Smitswinkel Bay | 0.218 | 0.463 | 0.010 | 0.024 |
| 15 | Pringle Bay | 0.218 | 0.489 | 0.044 | 0.137 |
| 16 | Franschhoek Pass | 0.172 | 0.465 | 0.000 | 0.000 |
| 17 | Greyton | 0.225 | 0.540 | 0.038 | 0.127 |
| 18 | Stanford | 0.139 | 0.373 | 0.013 | 0.032 |
| 19 | Agulhas | 0.223 | 0.557 | 0.003 | 0.007 |
| 20 | Kogmanskloof Pass | 0.157 | 0.358 | 0.000 | 0.000 |
| 21 | Bontebok | 0.195 | 0.476 | 0.029 | 0.080 |
| 22 | DeHoop | 0.190 | 0.494 | 0.000 | 0.000 |
| 23 | Anysberg | 0.185 | 0.525 | 0.010 | 0.025 |
| 24 | Tradouws Pass | 0.218 | 0.602 | 0.016 | 0.033 |
| 25 | Heidelberg | 0.311 | 0.726 | 0.016 | 0.053 |
| 26 | Garcia Pass | 0.177 | 0.436 | 0.006 | 0.013 |
| 27 | Still Bay | 0.180 | 0.403 | 0.054 | 0.169 |
| 28 | Albertinia | 0.180 | 0.432 | 0.003 | 0.007 |
| 29 | Vlees Bay | 0.210 | 0.490 | 0.048 | 0.100 |
| 30 | Mossel Bay | 0.154 | 0.382 | 0.006 | 0.013 |
| 31 | Seweweekspoort | 0.190 | 0.441 | 0.006 | 0.021 |
| 32 | Rooiberg Pass | 0.139 | 0.357 | 0.006 | 0.013 |
| 33 | Cloete Pass | 0.202 | 0.461 | 0.025 | 0.063 |
| 34 | Swartberg Pass | 0.228 | 0.567 | 0.013 | 0.031 |
| 35 | Robinsons Pass | 0.185 | 0.432 | 0.000 | 0.000 |
| 36 | Grootbrak | 0.142 | 0.369 | 0.000 | 0.000 |
| 37 | Outeniqua Pass | 0.152 | 0.373 | 0.016 | 0.049 |
| 38 | Daskop | 0.167 | 0.447 | 0.019 | 0.049 |
| 39 | Blesberg | 0.157 | 0.416 | 0.006 | 0.013 |
| 40 | Potjiesberg Pass | 0.167 | 0.389 | 0.013 | 0.039 |
| 41 | Plettenberg Bay | 0.127 | 0.307 | 0.006 | 0.019 |
| 42 | Misgund | 0.149 | 0.384 | 0.054 | 0.183 |
| 43 | Kougaberg | 0.256 | 0.635 | 0.010 | 0.031 |
| 44 | Joubertina | 0.197 | 0.491 | 0.013 | 0.043 |
| 45 | Nooitgedacht | 0.190 | 0.460 | 0.029 | 0.075 |
| 46 | Assegaaibos | 0.180 | 0.436 | 0.035 | 0.092 |
| 47 | East Baviaanskloof | 0.195 | 0.441 | 0.013 | 0.032 |
| 48 | Humansdorp | 0.205 | 0.616 | 0.000 | 0.000 |
| 49 | Stinkhoutberg | 0.180 | 0.460 | 0.016 | 0.037 |
| 50 | Suurberg Pass | 0.144 | 0.379 | 0.032 | 0.076 |
| 51 | Grahamstown | 0.152 | 0.412 | 0.006 | 0.021 |
|  | Overall | 1.559 | 0.583 | 0.298 | 0.230 |

Note: Estimates of per nucleotide site diversity (θ) were generated for each of the cpDNA and nuDNA datasets and for each locale using the average number of pairwise nucleotide differences among all sequences (“Theta-pi”) and the number of polymorphic sites for all sequences (“Theta-S”) after Tajima (1989).

**Table S4. Population graph “social network” node parameters**

| Dataset | locale | closeness | betweenness | degree | centrality |
| --- | --- | --- | --- | --- | --- |
| chloroplast | 1 | 0.0004 | 0 | 1 | 0.0008 |
|  | 2 | 0.0004 | 47 | 2 | 0.0130 |
|  | 3 | 0.0003 | 0 | 2 | 0.0524 |
|  | 4 | 0.0003 | 0 | 2 | 0.0675 |
|  | 5 | 0.0004 | 40 | 4 | 0.6866 |
|  | 6 | 0.0003 | 92 | 4 | 0.1525 |
|  | 7 | 0.0003 | 46 | 5 | 1.0000 |
|  | 8 | 0.0003 | 16 | 4 | 0.8453 |
|  | 9 | 0.0003 | 0 | 4 | 0.8871 |
|  | 10 | 0.0004 | 321 | 4 | 0.5431 |
|  | 11 | 0.0004 | 115 | 5 | 0.9121 |
|  | 12 | 0.0003 | 0 | 2 | 0.5671 |
|  | 13 | 0.0004 | 205 | 3 | 0.0599 |
|  | 14 | 0.0005 | 592 | 4 | 0.2497 |
|  | 15 | 0.0004 | 246 | 6 | 0.3031 |
|  | 16 | 0.0004 | 26 | 4 | 0.0662 |
|  | 17 | 0.0004 | 29 | 5 | 0.3405 |
|  | 18 | 0.0004 | 236 | 6 | 0.0873 |
|  | 19 | 0.0004 | 284 | 4 | 0.0751 |
|  | 20 | 0.0003 | 0 | 2 | 0.0654 |
|  | 21 | 0.0003 | 10 | 3 | 0.0539 |
|  | 22 | 0.0004 | 24 | 4 | 0.0662 |
|  | 23 | 0.0004 | 63 | 5 | 0.1227 |
|  | 24 | 0.0004 | 0 | 1 | 0.0000 |
|  | 25 | 0.0004 | 25 | 3 | 0.0577 |
|  | 26 | 0.0004 | 0 | 1 | 0.0000 |
|  | 27 | 0.0003 | 0 | 1 | 0.0003 |
|  | 28 | 0.0004 | 1 | 2 | 0.0035 |
|  | 29 | 0.0003 | 47 | 2 | 0.0009 |
|  | 30 | 0.0003 | 92 | 2 | 0.0137 |
|  | 31 | 0.0004 | 151 | 3 | 0.0288 |
|  | 32 | 0.0003 | 0 | 2 | 0.0006 |
|  | 33 | 0.0004 | 0 | 2 | 0.0312 |
|  | 34 | 0.0004 | 455 | 4 | 0.0299 |
|  | 35 | 0.0004 | 380 | 3 | 0.0055 |
|  | 36 | 0.0003 | 0 | 1 | 0.0017 |
|  | 37 | 0.0004 | 47 | 3 | 0.0065 |
|  | 38 | 0.0003 | 12 | 3 | 0.0571 |
|  | 39 | 0.0005 | 595 | 4 | 0.0782 |
|  | 40 | 0.0003 | 92 | 3 | 0.0060 |
|  | 41 | 0.0003 | 0 | 2 | 0.0006 |
|  | 42 | 0.0003 | 0 | 2 | 0.0004 |
|  | 43 | 0.0002 | 0 | 2 | 0.0000 |
|  | 44 | 0.0002 | 92 | 3 | 0.0001 |
|  | 45 | 0.0002 | 0 | 2 | 0.0000 |
|  | 46 | 0.0003 | 138 | 4 | 0.0002 |
|  | 47 | 0.0003 | 52 | 4 | 0.0002 |
|  | 48 | 0.0003 | 0 | 2 | 0.0005 |
|  | 49 | 0.0003 | 0 | 1 | 0.0000 |
|  | 50 | 0.0003 | 240 | 3 | 0.0004 |
|  | 51 | 0.0003 | 352 | 4 | 0.0016 |
|  |  |  |  |  |  |
| nuclear | 1 | 0.0004 | 0 | 1 | 0.9800 |
|  | 2 | 0.0004 | 3 | 2 | 0.8965 |
|  | 3 | 0.0004 | 0 | 1 | 0.0000 |
|  | 4 | 0.0004 | 0 | 0 | 0.2733 |
|  | 5 | 0.0004 | 5 | 3 | 1.0000 |
|  | 6 | 0.0004 | 0 | 1 | 0.8513 |
|  | 7 | 0.0004 | 0 | 0 | 0.2733 |
|  | 8 | 0.0004 | 0 | 1 | 0.0000 |
|  | 9 | 0.0004 | 0 | 1 | 0.0000 |
|  | 10 | 0.0004 | 0 | 1 | 0.5310 |
|  | 11 | 0.0004 | 0 | 0 | 0.2733 |
|  | 12 | 0.0004 | 1 | 2 | 0.0000 |
|  | 13 | 0.0004 | 0 | 0 | 0.2733 |
|  | 14 | 0.0004 | 0 | 0 | 0.2733 |
|  | 15 | 0.0004 | 0 | 0 | 0.2733 |
|  | 16 | 0.0004 | 0 | 0 | 0.2733 |
|  | 17 | 0.0004 | 0 | 0 | 0.2733 |
|  | 18 | 0.0004 | 1 | 2 | 0.0000 |
|  | 19 | 0.0004 | 0 | 0 | 0.2733 |
|  | 20 | 0.0004 | 0 | 0 | 0.2733 |
|  | 21 | 0.0004 | 0 | 1 | 0.4782 |
|  | 22 | 0.0004 | 0 | 0 | 0.2733 |
|  | 23 | 0.0004 | 0 | 1 | 0.0000 |
|  | 24 | 0.0004 | 0 | 0 | 0.2733 |
|  | 25 | 0.0004 | 0 | 0 | 0.2733 |
|  | 26 | 0.0004 | 0 | 1 | 0.0000 |
|  | 27 | 0.0004 | 0 | 1 | 0.0000 |
|  | 28 | 0.0004 | 0 | 0 | 0.2733 |
|  | 29 | 0.0004 | 0 | 1 | 0.0000 |
|  | 30 | 0.0004 | 0 | 1 | 0.0000 |
|  | 31 | 0.0004 | 0 | 1 | 0.0000 |
|  | 32 | 0.0004 | 0 | 1 | 0.0000 |
|  | 33 | 0.0004 | 0 | 1 | 0.0000 |
|  | 34 | 0.0004 | 0 | 1 | 0.0000 |
|  | 35 | 0.0004 | 0 | 0 | 0.2733 |
|  | 36 | 0.0004 | 0 | 1 | 0.0289 |
|  | 37 | 0.0004 | 0 | 0 | 0.2733 |
|  | 38 | 0.0004 | 0 | 0 | 0.2733 |
|  | 39 | 0.0004 | 0 | 0 | 0.2733 |
|  | 40 | 0.0004 | 0 | 0 | 0.2733 |
|  | 41 | 0.0004 | 0 | 1 | 0.0000 |
|  | 42 | 0.0004 | 0 | 1 | 0.0000 |
|  | 43 | 0.0004 | 0 | 1 | 0.5742 |
|  | 44 | 0.0004 | 0 | 1 | 0.0000 |
|  | 45 | 0.0004 | 0 | 1 | 0.0000 |
|  | 46 | 0.0004 | 0 | 0 | 0.2733 |
|  | 47 | 0.0004 | 1 | 2 | 0.0000 |
|  | 48 | 0.0004 | 0 | 0 | 0.2733 |
|  | 49 | 0.0004 | 0 | 0 | 0.2733 |
|  | 50 | 0.0004 | 0 | 1 | 0.0000 |
|  | 51 | 0.0004 | 1 | 2 | 0.4122 |

Note: see Table S1 for details on locales, and Materials and Methods section for details on parameters.

**Table S5. Generalized Linear Mixed Model (GLMM) analysis of landscape variables**


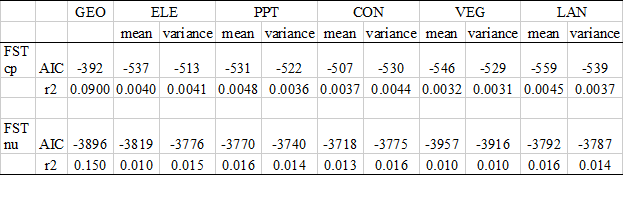


Note: GLMM analyses using locale pairwise FST for chloroplast (cp) and nuclear (nu) genetic datasets, with Akaike Information Criterion (AIC) and Pearson's r^2^ presented for each variable of GEO: Euclidean Distance; ELE: Elevation; PPT: Annual rainfall; CON: Rainfall concentration; VEG: Vegetation type; LAN: Land-use type. For details, see Materials and Methods section.

**Figure S1.** Raster landscape raster maps of southern South Africa fit to the 51 sampled locales of *Leucadendron salignum* (shown as “white” dots, see Figure 1 and Table S1 for sampled locale information). Landscape variables shown for (a) elevation; (b) annual rainfall; (c) seasonal rainfall concentration; (d) vegetation type; (e) land-use type. For details, see Materials and Methods section.


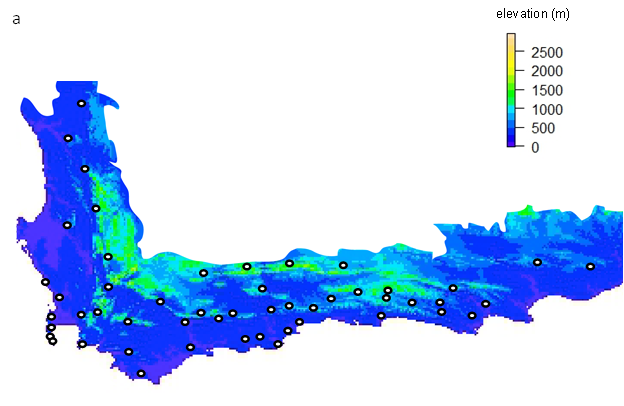

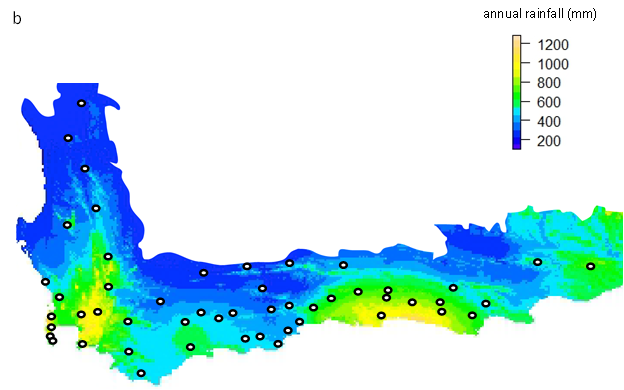


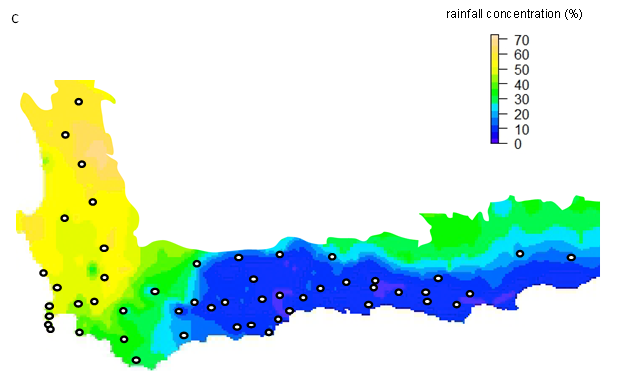


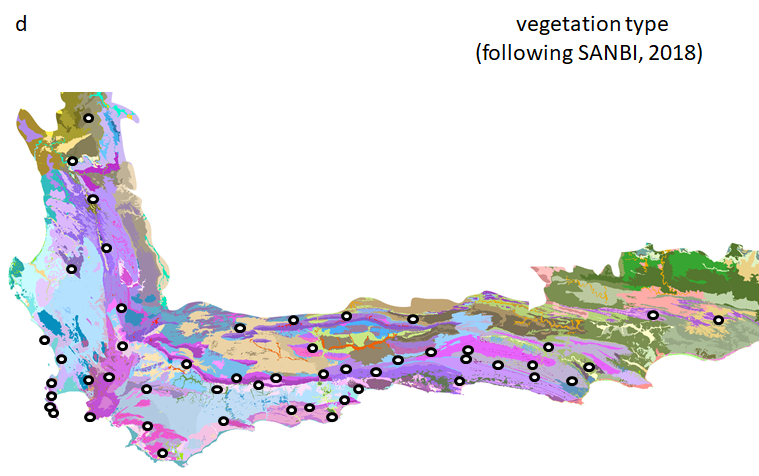

**Figure S2.** Matrix of pairwise F_ST_ between locales for *Leucadendron salignum* chloroplast (cpDNA; top) and nuclear (nuDNA; bottom) SNP variation. Values >0.25 for cpDNA are significant (p<0.01), which are 1241 of the 1275 pairwise comparisons. Values >0.10 for nuDNA are significant (p<0.01), which are 557 of the 1275 pairwise comparisons. For details on analyses, see Materials and Methods section. For locale information, see Table S1.

**
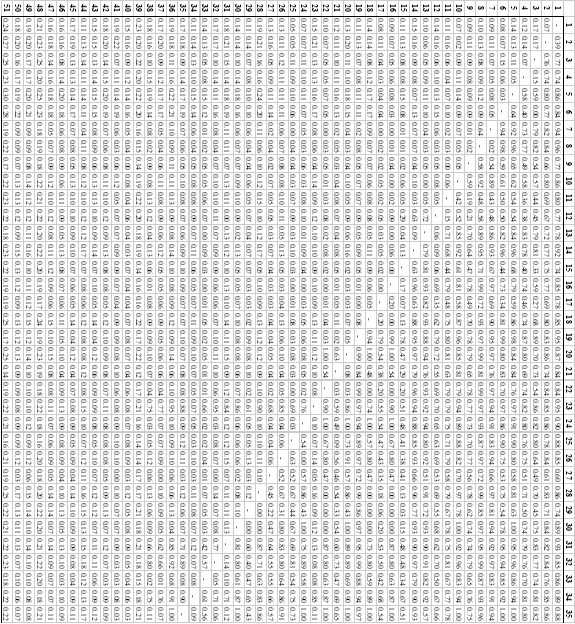
**

**
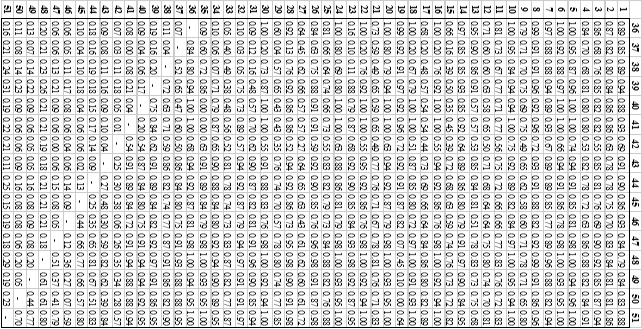
**

**Figure S3.** fastSTRUCTURE (Raj et al., 2014) analysis of *Leucadendron salignum* nuclear DNA data from 51 sampled locales (see Figure 1 for geographic sampling, Table S1 for locale information). As noted in the Materials and Methods, there were four individuals sampled per locale, for a total of 204 diploid individuals and 408 nucleotide sequences. Results are shown for locale clustering under the model test of K=2.

**Figure S4.** Topographic map of southern South Africa with labeled features of relevance. Dotted line denotes recognized Cape Floristic Region.

**
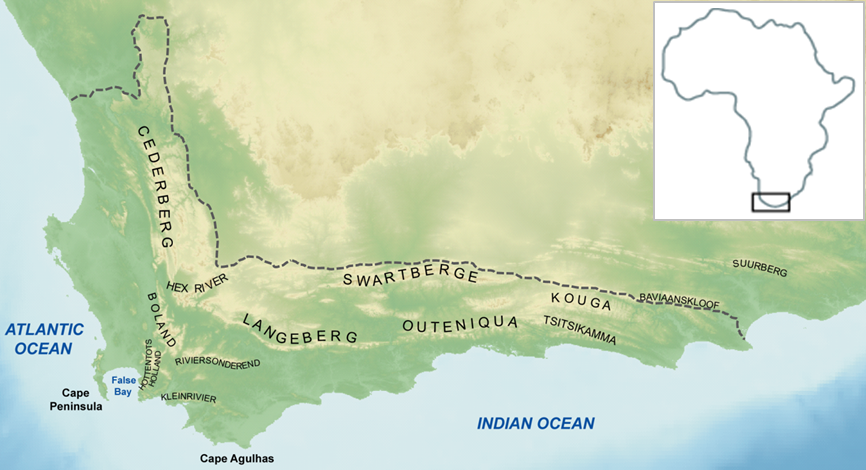
**

**Figure S5.** Popgraph of genetic connectivity among 51 sampled locales of *Leucadendron salignum* nuclear DNA (see Figure 1 for geographic sampling, Table S1 for locale information).

**Figure S6.** Distributions of the permutation analyses of associations between popgraph genetic connections among locales and landscape variables measured between locales. For each graph, the “red” plotted point reflects where the observed mean or variance of the popgraph landscape variable falls in comparison to the permuted distribution of the landscape variable mean or variance. Significance indicates that the observed landscape variable between locales was more or less associated with popgraph genetic connections between locales than predicted by the permutation analysis. The landscape variables are shown as (a) mean elevation (p<0.001), and variance in elevation (p<0.001); (b) mean annual rainfall (p=0.026), and variance in annual rainfall (p=0.008); (c) mean seasonal rainfall concentration (p=0.020), and variance in seasonal rainfall concentration (p<0.001); (d) mean vegetation type (p=0.449), and variance in vegetation type (p=0.140); (e) mean human land-use type (p=0.016), and variance in human land-use type (p=0.077). For details, see Materials and Methods section.

**
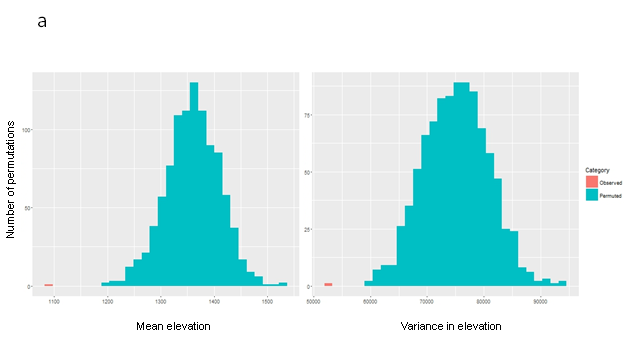
**

**
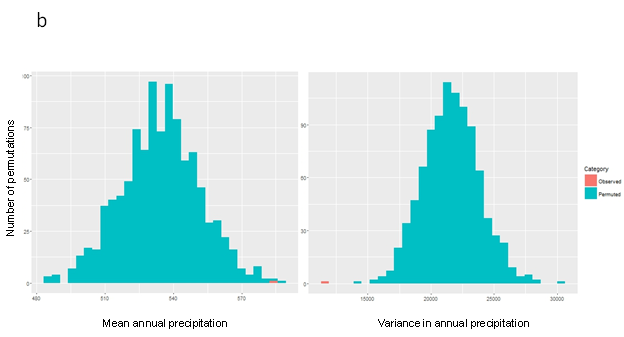
**

**
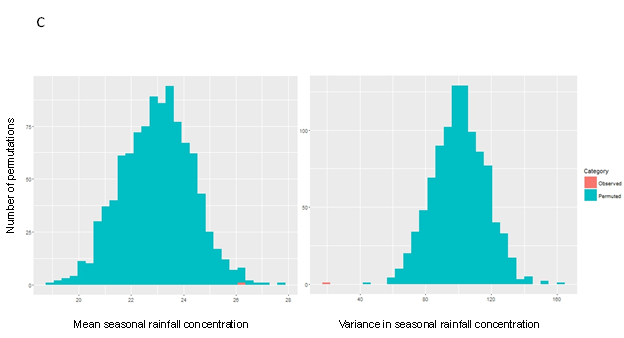
**

**
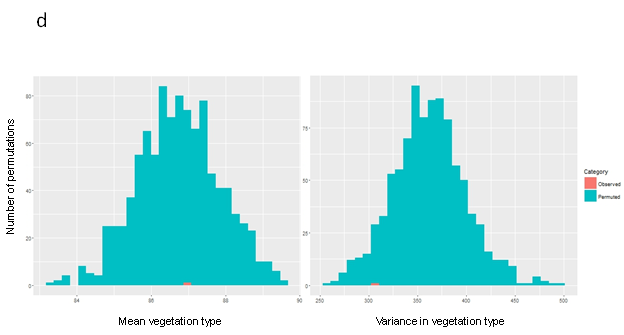
**

**
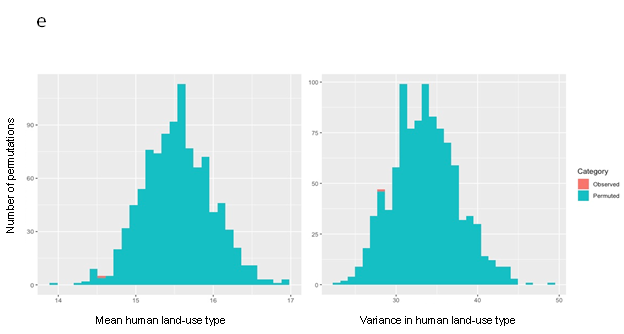
**

**References**

Barker, N.P., Vanderpoorten, A., Morton, C. M, & Rourke, J. P. (2004). Phylogeny, biogeography, and the evolution of life-history traits in Leucadendron (Proteaceae). Molecular Phylogenetics and Evolution, 33, 845-860.

Bergh, N. G., Hedderson, T. A., Linder, H. P., & Bond, W. J. (2007). Palaeoclimate-induced range shifts may explain current patterns of spatial genetic variation in renosterbos. Taxon, 56, 393-408.

Birky, C. W. Jr. (2001). The inheritance of genes in mitochondria and chloroplasts: laws, mechanisms, and models. Annual Review of Genetics, 35, 125-148.

Drummond, A. J., & Rambaut, A. (2007). BEAST: Bayesian evolutionary analysis by sampling trees. BMC Evolutionary Biology, 7, 214.

Hoffmann, V., Verboom, G. A., & Cotterill, F. P. D. (2015). Dated plant phylogenies resolve Neogene climate and landscape evolution in the Cape Floristic Region. PLoS ONE, 10, e0137847.

Hu, J., Lavin, M., Wojciechowski, M., & Sanderson, M. (2000). Phylogenetic systematics of the tribe Millettieae (Leguminosae) based on chloroplast trnK/matK sequences and its implications for evolutionary patterns in Papilionoideae. American Journal of Botany, 87, 418-430.

Kearse, M., Moir, R., Wilson, A., Stones-Havas, S., Cheung, M., Sturrock, S., Buxton, S., Cooper, A., Markowitz, S., Duran, C., Thierer, T., Ashton, B., Meintjes, P., & Drummond, A. (2012). Geneious Basic: an integrated and extendable desktop software platform for the organization and analysis of sequence data. Bioinformatics, 28, 1647–1649.

Pharmawati, M., Yan, G., & Finnegan, P. M. (2005). Molecular variation and fingerprinting of Leucadendron cultivars (Proteaceae) by ISSR markers. Annals of Botany, 95, 1163-1170.

Posada, D. (2008). jModelTest: Phylogenetic model averaging. Molecular Biology and Evolution, 25, 1253–1256.

Rambaut, A. (2012). FigTree. Retrieved from http://tree.bio.ed.ac.uk/software/figtree/

Sauquet, H., Weston, P. H., Barker, N. P., Anderson, C. L., Cantrill, D. J., & Savolainen, V. (2009). Using fossils and molecular data to reveal the origins of the Cape Proteas (Subfamily Proteoideae). Molecular Phylogenetics and Evolution, 51, 31-43.

Tajima, F. (1989). Statistical‐method for testing the neutral mutation hypothesis by DNA

polymorphism. Genetics, 123, 585–595.

Tonnabel, J., Mignot, A., Douzery, E. J., Rebelo, A. G., Schurr, F. M., Midgley, J., Illing, N., Justy, F., Orcel, D., & Olivieri, I. (2014). Convergent and correlated evolution of major life-history traits in the angiosperm genus Leucadendron (Proteaceae). Evolution, 68, 2775-2792.

Valente, L. M., Reeves, G., Schnitzler, J., Mason, I. P., Fay, M. F., Rebelo, A. G., & Barraclough, T. G. (2010). Diversification of the African genus Protea (Proteaceae) in the Cape biodiversity hotspot and beyond: equal rates in different biomes. Evolution, 64, 745-759.

Wolfe, K. H., Li, W. H., & Sharp, P. M. (1987) Rates of nucleotide substitution vary greatly among plant mitochondrial, chloroplast, and nuclear DNAs. Proceedings of the National Academy of Sciences USA, 84, 9054-9058.
